# Sensitivity analysis for reporting bias on the time-dependent summary receiver operating characteristics curve in meta-analysis of prognosis studies with time-to-event outcomes

**DOI:** 10.1017/rsm.2025.14

**Published:** 2025-03-21

**Authors:** Yi Zhou, Ao Huang, Satoshi Hattori

**Affiliations:** 1 Beijing International Center for Mathematical Research, Peking University, Beijing, China; 2 Department of Biomedical Statistics, Graduate School of Medicine, Osaka University, Osaka, Japan; 3 Graduate School of Human Development and Environment, Kobe University, Kobe, Japan; 4 Department of Medical Statistics, University Medical Center Göttingen, Göttingen, Germany; 5 Integrated Frontier Research for Medical Science Division, Institute for Open and Transdisciplinary Research Initiatives (OTRI), Osaka University, Osaka, Japan

**Keywords:** meta-analysis of prognosis studies, reporting bias, sensitivity analysis, time-dependent SROC curve

## Abstract

In prognosis studies with time-to-event outcomes, the survivals of groups with high/low biomarker expression are often estimated by the Kaplan–Meier method, and the difference between groups is measured by the hazard ratios (HRs). Since the high/low expressions are usually determined by study-specific cutoff values, synthesizing only HRs for summarizing the prognostic capacity of a biomarker brings heterogeneity in the meta-analysis. The time-dependent summary receiver operating characteristics (SROC) curve was proposed as a cutoff-free summary of the prognostic capacity, extended from the SROC curve in meta-analysis of diagnostic studies. However, estimates of the time-dependent SROC curve may be threatened by reporting bias in that studies with significant outcomes, such as HRs, are more likely to be published and selected in meta-analyses. Under this conjecture, this paper proposes a sensitivity analysis method for quantifying and adjusting reporting bias on the time-dependent SROC curve. We model the publication process determined by the significance of the HRs and introduce a sensitivity analysis method based on the conditional likelihood constrained by some expected proportions of published studies. Simulation studies showed that the proposed method could reduce reporting bias given the correctly-specified marginal selection probability. The proposed method is illustrated on the real-world meta-analysis of Ki67 for breast cancer.

## Highlights

### What is already known

- Heterogeneous cutoff values over studies often occur in meta-analysis examining the association of a continuous biomarker with a binary outcome (diagnostic study) and with a time-to-event outcome (prognostic study).
- For diagnostic meta-analysis, the summary receiver operating characteristics (SROC) curve and its area under the curve are widely recognized as a useful tool and used to handle the issue of heterogeneous cutoff values.
- For prognostic meta-analysis with time-to-event outcomes, the time-dependent SROC method, which is an extension of the SROC method in diagnostic meta-analysis, has been developed by Hattori and Zhou (2016, *Statistics in Medicine* 35(26), 4746–4763) and is useful to address the issue of heterogeneous cutoff values.
- In meta-analysis, large studies or studies with significant results are more likely to be published and observed for meta-analysis; synthesizing only these studies may result in reporting bias, also known as, publication bias or small-study effects.
- Reporting bias is unavoidable and may also induce biased estimates of the time-dependent SROC method by the bivariate normal model of Hattori and Zhou (2016, *Statistics in Medicine* 35(26), 4746–4763).

### What is new

- For prognostic studies with time-to-event outcomes, the selective publication process of studies and the selective reporting of the Kaplan–Meier estimates can be influenced by the significance of the hazard ratios, equivalently, the significance of the log-rank tests.
- To model selective publication processes driven by the log-rank test, inference of trivariate model has been successfully established, which is essentially beyond the sensitivity analysis method for reporting bias in meta-analysis of diagnostic studies.
- The proposed model was constrained by the marginal selection probability as a sensitivity parameter; the impact of reporting bias on the time-dependent SROC method can be evaluated by varying the marginal selection probability within (0,1).

### Potential impact for Research Synthesis Methods readers outside the authors’ field

- We proposed the first method dealing with reporting bias on the time-dependent SROC method for meta-analysis of prognosis studies with time-to-event outcomes; then, users could evaluate the robustness of the estimates when evaluating the prognostic capacity of the biomarker.
- By incorporating the ability to address reporting bias, our method enhanced the utility and applicability of the time-dependent SROC method of Hattori and Zhou (2016, *Statistics in Medicine* 35(26), 4746–4763) for meta-analysis of prognosis studies.

## 1 Introduction

Biomarkers have been playing critical roles in medical therapeutics and precision medicine, and many clinical studies aim to investigate the associations between biomarkers and subjects’ outcomes. Usually, diagnostic or prognostic capacity is measured by the associations between subjects’ biomarker expression values and their (binary) disease outcomes or time-to-event outcomes, respectively. Correspondingly, studies evaluating diagnostic or prognostic capacity of the biomarker of interest are referred to as diagnostic or prognosis studies, respectively. In this paper, we are interested in prognosis studies that evaluate the association between a continuous biomarker and time-to-event outcomes; although prognosis studies can have continuous or binary outcomes, we focus on studies with time-to-event outcomes.

In the analysis of a prognosis or diagnostic study with a continuous biomarker, subjects are often classified into the high/low expression (or positive/negative) groups by a certain cutoff value. In a diagnostic study, diagnostic capacity of the biomarker is usually represented by the pair of sensitivity and specificity estimated at a specified cutoff value.^1^ More informative measurements include the receiver operator characteristic (ROC) curve and the area under the curve (AUC) since they present the diagnostic capacity over the range of cutoff value. This successful ROC methodology, developed for diagnostic studies with binary outcomes, has been successfully extended to time-to-event outcomes. By formulating the problem as diagnosing whether a subject survives beyond a fixed time point, such as one year, the time-dependent sensitivity and specificity are defined. Then, the time-dependent ROC curve and its AUC are utilized for evaluating prognostic capacity on time-to-event outcomes.^2^ ^,^ ^3^

Meta-analysis is a useful tool to synthesize data of multiple studies and provides a more precise estimate of diagnostic or prognostic capacity. In meta-analysis of diagnostic studies, study-specific cutoff values induce correlation between the empirical sensitivity and specificity pairs among the collected studies. Additionally, they bring difficulty in interpretation of the meta-analytical results that are aggregated by the standard meta-analysis technique, such as the random-effects model. Thus, bivariate models are recommended to model the empirical sensitivity and specificity pairs and the summary ROC (SROC) curve^4^ ^–^ ^6^ to show diagnostic capacity. The SROC curve presents the monotonic relationship between sensitivity and 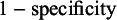 at all possible cutoff values, and the area under the SROC curve, namely the summary AUC (SAUC), gives a univariate summary of diagnostic capacity. Properties and extensions of the SROC curve have been much discussed in many statistical literature, and the SROC curve and the SAUC are widely used as the main results in meta-analysis of diagnostic studies.^1^ In contrast, in clinical journals, almost all meta-analysis of prognosis studies aggregate the hazard ratios (HRs) using the standard meta-analysis technique without caring about the study-specific cutoff values among studies.^7^ ^–^ ^11^ The major problem is that varying cutoff values contribute to large heterogeneity in the meta-analysis results.^7^ ^,^ ^8^ A couple of papers proposed methods to aggregate the HRs accounting for heterogeneous cutoff values.^12^ ^,^ ^13^ However, these HR-based summary measures are hard to interpret. Motivated by the wide acceptance of the SROC curves in meta-analysis of diagnostic studies, meta-analytic version of the time-dependent ROC curve was developed, that is, the time-dependent SROC curve, denoted by 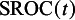 . Comparing to the synthesis of the HRs, 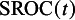 provides a visual presentation of the overall prognostic capacity without depending on one or multiple specific cutoff values. Additionally, 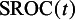 synthesizes prognostic capacity at specific time points; thus, one could view the change of prognostic capacity over time. The area under 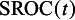 , denoted by 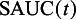 , is useful to quantify prognostic capacity. By testing the null hypothesis that 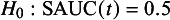 , one could examine whether or not the biomarker has significant prognostic capacity. To make inference about 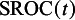 , Combescure et al.^14^ employed the non-linear mixed model to model the biomarker and time-to-event distributions. Hattori and Zhou^15^ proposed the bivariate normal model and the bivariate binomial model by extending the bivariate models^4^ ^,^ ^5^ for meta-analysis of diagnostic studies. All these methods utilize the Kaplan–Meier (KM) estimates extracted from literatures. Among the models estimating 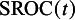 , the bivariate normal model of Hattori and Zhou^15^ (hereinafter, the HZ model) appears to be simplest in the inference and practical implementation. As proposed, the pairs of empirical time-dependent sensitivity and specificity with their variances are estimated using the retrieved number of patients and KM estimates at several time points. Then, the HZ model bivariately models the empirical time-dependent sensitivity and specificity pairs for estimating 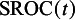 . The detailed inference procedure is introduced in Section 3.

Despite the usefulness of meta-analyses, validity of the synthesized results is often threatened by publication bias, also known as small-study effects. Publication bias is induced by selective publication, where large studies or studies with significant outcomes are more likely to be published and collected for meta-analysis, and consequently, overlooking unpublished studies in meta-analysis can lead to biased estimates and overoptimistic conclusions.^16^ In univariate meta-analysis of intervention studies (e.g., randomized clinical trials), methods for assessing and adjusting publication bias have been intensively studied. Despite the popular graphical methods (e.g., the funnel plot and the trim-and-fill method), sensitivity analysis methods with selection functions, including the Heckman-type selection functions,^17^ ^,^ ^18^ the *t*-statistic based selection function,^19^ and the worst-case analysis,^20^ provide more careful evaluations on the impact of publication bias. In meta-analysis of diagnostic studies, several selection function based methods have been proposed for dealing with publication bias on the estimate of the SROC curve. Most methods modeled the selective publication process by the Heckman-type selection functions.^21^ ^–^ ^23^ Recently, Zhou et al.^24^ introduced the cutoff-dependent selection function, which is the probit model on the *t*-type statistic of the empirical sensitivity and specificity pairs. Specifically, the *t*-type statistic is defined by that of the linear combination of the logit-transformed empirical sensitivities and specificities. Thus, the cutoff-dependent selection function can model a variety of selective publication processes determined by the significance of sensitivity, specificity, or both. Since the scientific arguments in each diagnostic study are mainly based on the cutoff-dependent quantities (e.g., sensitivity, specificity, and their derivatives), such cutoff-dependent selection functions would be more appealing to model the selective publication.

Publication bias can also affect the estimate of 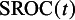 . Furthermore, 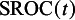 may suffer from additional source of bias. As aforementioned, inference of 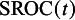 relies on the KM estimates extracted from the literature, where authors might place the figures of KM estimates to highlight their findings of substantial survival improvement. However, not all the literatures report the figures of KM estimates. Exclusion of studies missing KM estimates may result in biased 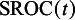 estimations, which is an issue of selective reporting.^25^ In this paper, we refer to the combined bias resulting from selective publication and selective reporting of KM estimates as *reporting bias*. Consequently, we aim to propose a sensitivity analysis method for assessing the impact of reporting bias on 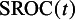 estimated by the HZ model. Due to the similarity between the HZ model and the bivariate normal model in meta-analysis of diagnostic studies, an intuitive idea is to apply the method of Zhou et al.^24^ to the HZ model. However, the publication mechanisms between diagnostic and prognosis studies are different; thus, the selection function defined in Zhou et al.^24^ may not be appealing to model the publication mechanism in prognosis studies. In prognostic studies, the KM estimates of the high/low expression groups are often reported graphically with the *P*-values of the log-rank test, and the conclusions about prognostic capacity of the biomarker are measured by the HRs. Thus, the significance of the log-rank test, or the significance of the log-transformed HR (lnHR) between two groups, is supposed to be a determinant of the publication of prognosis studies. On the other hand, publication mechanism modeled by the selection function on the test statistic is more interpretable. These motivated us to propose the sensitivity analysis method that employs the test statistic based selection function^24^ to address reporting bias in meta-analysis of prognosis studies. However, it is challenging in the development. In Zhou et al.,^24^ the *t*-type statistic can be expressed by sensitivity and specificity, which are reported as outcomes in the diagnostic studies. They successfully extended the likelihood-based sensitivity analysis method of Copas^19^ into the bivariate normal model for the logit-transformed sensitivity and specificity. In meta-analysis of prognosis studies, the data of time-dependent sensitivity and specificity pairs are not observable outcomes, and it is difficult to re-express the log-rank statistic (equivalently, the *t*-statistic of lnHR without considering covariates) as a linear function of the time-dependent sensitivity and specificity pairs. To overcome this, we propose a trivariate model for logit-transformed time-dependent sensitivity and specificity and the lnHR. Based on the trivariate model, the conditional likelihood taking into account the selection function of the log-rank statistic is derived. The conditional likelihood is further constrained by the marginal selection probability. By specifying a plausible range of marginal selection probabilities, one could assess the possibly minimal and maximal impact of reporting bias on 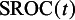 and 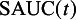 . This paper provides an idea for dealing with reporting bias in meta-analysis of studies with time-to-event outcomes.

The rest of this article is organized as follows. In Section 2, we introduce the issue of reporting bias using a motivating meta-analysis of Ki67. In Section 3, we describe the general data structure and review the HZ model without taking into account selective publication. In Section 4, we propose the trivariate model and the sensitivity analysis method for reporting bias in detail. In Section 5, we revisit the meta-analysis of Ki67 and evaluate the potential impact of reporting bias by the proposed method. In Section 6, simulation studies are conducted to evaluate the performance of the proposed method. In Section 7, we conclude this work with a discussion.

**Table 1:** Scenarios of distributions of biomarker and cutoff values used in simulation studies. *e* follows the standard logistic distribution

	Biomaker ( 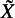 )		
Scenario	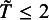	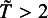	Cutoff value
1	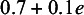	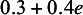	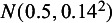
2	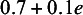	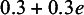	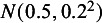
3	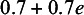	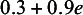	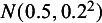
4	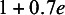	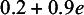	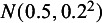
5	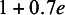	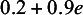	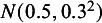
6	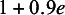	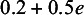	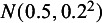

**Figure 1:**
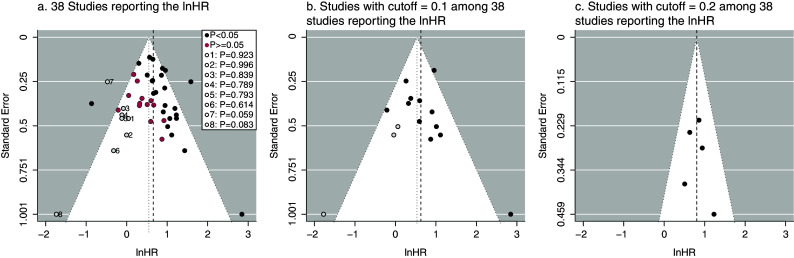
The funnel plot and the trim and fill method for detecting reporting bias in meta-analysis of Ki67. The vertical black dashed lines are the integrated lnHRs without considering reporting bias. The central axes of the funnel plots are the adjusted lnHRs. The open circle points are the filled unpublished studies. The red and black points are the published studies categorized by the *P*-values of the lnHRs. P for the open circle point in the legends indicated the estimated *P*-values of the imputed lnHRs of the filled studies.

## 2 Motivating example: reporting bias in meta-analysis of Ki67

De Azambuja et al.^8^ conducted meta-analysis to evaluate the prognostic capacity of Ki67. They synthesized the reported HRs that assessed the association between Ki67 and the survival outcome of patients with early breast cancer. In the overall survival outcomes, 38 studies reported the HRs (or equivalently, the log-transformed HRs, denoted by lnHRs) with the corresponding standard errors (SEs). The data are presented in Table 1 of De Azambuja et al.,^8^ where the SEs can be derived from the 95% confidence intervals (CIs). The HR was defined by that between the high versus low expression of Ki67 groups, and the high/low expressions were decided by various study-specific cutoff values, ranging from 0.035 to 0.286. The heterogeneous cutoff values contributed to some of the heterogeneity in this meta-analysis of the lnHRs.

The major concern, which is often too overlooked, is reporting bias, the mixture of selective publication of studies and selective reporting of outcomes. In this meta-analysis, the 38 studies may have been selectively published from the population of studies due to the significant results. Since the selective publication process of studies cannot be verified from the observed data, to explore the existence of potentially unpublished studies, one straightforward way is to use funnel plot of the lnHRs for visualization. In this meta-analysis, the unpublished studies were imputed by the trim-and-fill method until the funnel plot on the lnHR was symmetric. In Figure 1a, the funnel plot and the trim-and-fill method implied that some studies with insignificant lnHRs might be unpublished (the circle points in the plot), and studies with insignificant lnHRs (i.e., *P*-value greater than 0.5) seemed vulnerable to unpublication. However, since the lnHRs were estimated dependent of the study-specific cutoff values, the funnel plot on the lnHRs could be misleading and influenced by the heterogeneity resulted from cutoff values. To mitigate the influence of heterogeneous cutoff values, we then apply the trim-and-fill method in the strata of cutoff values. In the funnel plots of the subgroup of cutoff value equal to 0.1 (Figure 1b), the asymmetry of funnel plots suggested some potential unpublished studies determined by the significance of lnHRs, and reporting bias seemed more likely to occur in the subgroup of cutoff value equal to 0.1. In contrast, in the subgroup of cutoff value equal to 0.2 (Figure 1c), selective publication was not detected.

To evaluate the prognostic capacity independent of cutoff values, Hattori and Zhou^15^ re-analyzed this meta-analysis by their proposed HZ model. They extracted the KM estimates of the high/low expression groups at several time points and estimated 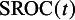 and 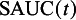 at the third ( 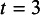 ) and the fifth ( 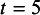 ) follow-up years. The estimate of SAUC(3) was 0.649 (95% CI: 0.606-0.690), and SAUC(5) was estimated as 0.646 (0.610, 0.680), indicating that Ki67 was still useful to discriminant patients with breast cancer at the third and fifth follow-up years. However, among the 38 studies, not all the studies reported the KM curves. The estimates of SAUC(3) and SAUC(5) were based on 23 and 21 studies, which reported the KM estimates at the third and fifth years, respectively.

**Figure 2:**
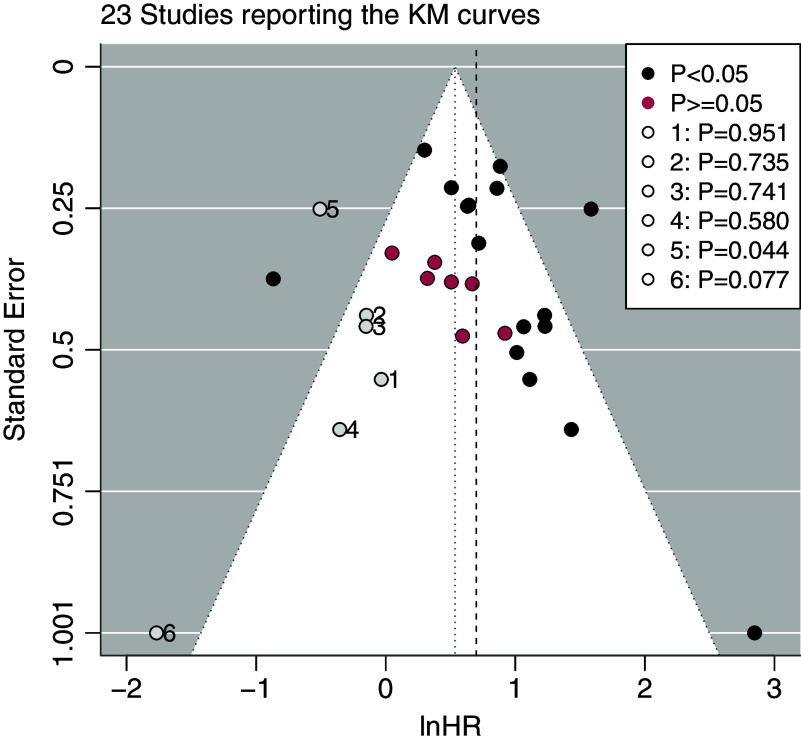
The funnel plot and the trim and fill method for detecting reporting bias in meta-analysis of Ki67 based on the 23 studies reporting the KM curves. The vertical black dashed lines are the integrated lnHRs without considering reporting bias. The central axes of the funnel plots are the adjusted lnHRs. The open circle points are the filled unpublished studies. The red and black points are the published studies categorized by the *P*-values of the lnHRs. P for the open circle point in the legends indicated the estimated *P*-values of the imputed lnHRs of the filled studies.

In summary, among the studies reporting the lnHRs, the trim-and-fill method detected some unpublished studies that were almost insignificant in the lnHRs (Figure 2). When using the HZ model for meta-analysis, less than 38 studies reporting KM estimates were used for synthesis. These two sources of missingness could lead to reporting bias in the results. Thus, to evaluate the robustness of the estimates of SROC(*t*) and SAUC(*t*), reporting bias including both selective publication of studies and selective reporting of the KM curves should be taken with caution. Although the funnel plot and the trim-and-fill method could raise some concerns about reporting bias in this meta-analysis, they are not useful for evaluating the potential impact of reporting bias on 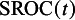 and 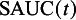 . To overcome this issue, we propose a sensitivity analysis method for evaluating the impact of reporting bias on 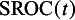 by the HZ model in the later sections.

## 3 Bivariate normal model without considering reporting bias

### 3.1 Notations and data structure

Suppose that *S* prognosis studies are conducted to evaluate the association between the expressions of biomarker and subjects’ time-to-event outcomes. Among the *S* studies, data of *N* prognosis studies are published and used in meta-analysis to summarize the prognostic capacity, while data of 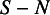 studies are missing. In this section, we do not consider the existence of reporting bias, that is, data of *N* published studies consist of the population or random sample from the *S* studies. Since we focus on the HZ model to synthesize data of the published studies, we follow the notations in Hattori and Zhou^15^ to introduce the latent individual patient data (IPD) in each study and the observable data for meta-analysis as follows.

Suppose that each prognosis study 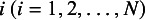 includes 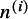 subjects, and the subjects are assumed to be random samples from the population of interest. For each subject, let 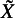 be the baseline measurement of biomarker, 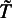 the failure time, 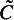 the right-censored time, and 
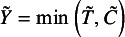
 the follow-up time. We use tilde to denote these variables for individuals, which are unextractable from the literature for meta-analysis. We assume that the distribution of 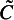 is identical across *N* studies as well as 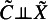 , and 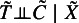 , where 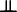 indicates variables are independent. These assumptions lead to 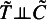 .

For each study *i*, let 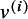 denote the study-specific cutoff value, which is not necessarily reported. This cutoff value separates subjects into the low expression group, denoted by 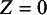 , if 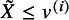 or the high expression group ( 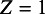 ) if 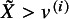 . The survival functions of the low and high expression groups are respectively denoted and defined by 

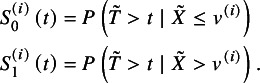

The disease status at time *t* is defined by the counting process 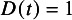 if 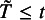 or 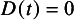 if 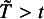 , indicating that subjects have an event before time *t* or survive after *t*, respectively. To facilitate the understanding, we showed the number of subjects given different disease statuses and expression groups in Table S1 in the Supplementary Material. Analogous to the diagnostic study, sensitivity and specificity at time *t* are respectively denoted and defined by 

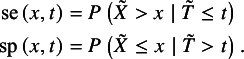



For meta-analysis, the following summarized data are required and can be extracted from published literatures. For study 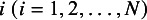 , let 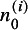 and 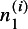 denote the number of subjects separated into the low and high expression groups, respectively; the total number of subjects is 
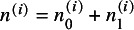
 . Let 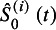 and 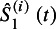 denote the KM estimates of the low and high expression groups, respectively, and they can be extracted from the reported plots of the KM curves at the partition of time interval 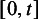 : 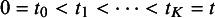 . Let 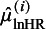 denote the reported lnHR between high versus low expression groups and 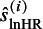 the corresponding SE; both are estimated by the Cox model.^26^ Among *N* studies, the sample medians of the follow-up time over the total subjects can also be extracted from some studies. These medians of follow-up time are used to estimate the censoring distribution in estimating the SEs of 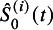 and 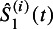 . (See Section 3.2 in Hattori and Zhou^15^ for more details.) We take the example of meta-analysis of Ki67 to illustrate the data structure with details explained in Section S1 of the Supplementary Material.

### 3.2 Bivariate normal model

In this section, we review the structure of HZ model and the definitions of 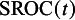 and 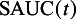 . The HZ model employs the bivariate normal-normal random-effects model to model the empirical pairs of logit-transformed time-dependent sensitivities and specificities, which are estimated using KM estimates from published literatures, allowing for heterogeneity from different sources (e.g., cutoff values, study designs, population, etc.) in time-dependent sensitivities and specificities across studies. It extended the model of Reitsma et al.^5^ which has been widely used in meta-analysis of diagnostic studies.

According to Hattori and Zhou,^15^ with the Bayes rule and simple algebraic manipulations, the true sensitivity and specificity at time *t* of study *i* can be respectively re-expressed by 
(1)

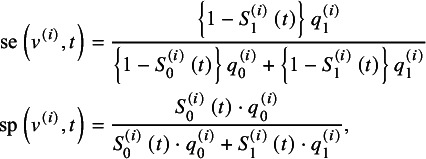

where 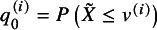 and 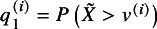 . With the data extracted from collected studies, 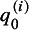 and 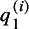 are estimated by 
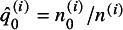
 and 
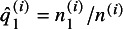
 , respectively; the time-dependent sensitivity and specificity in equation (1) are consistently estimated by substituting 
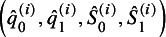
 for 
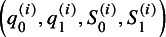
 . The resulting consistent estimators are denoted by 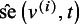 and 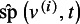 , respectively.

The HZ model, following Reitsma et al.,^5^ employs a logit-transformation to map the time-dependent sensitivity and specificity to the real number line 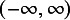 . While other transformations are also applicable,^27^ logit-transformation is widely accepted. Let 
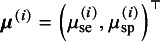
 denote the true time-dependent sensitivity and specificity pair of study *i* on the logit scale, that is, 

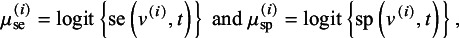

where 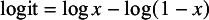 . At the between-study level, it is assumed that 
(2)

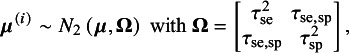

where 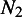 denotes the bivariate normal distribution, and 
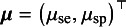
 is the overall mean at time *t* across multiple prognosis studies; 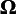 is the between-study variance-covariance matrix of 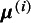 , where 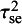 and 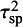 are the variance of 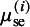 and 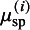 , respectively, and 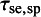 the covariance between them.

Let 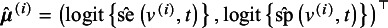 denote the consistent estimates of 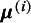 in each study. Hattori and Zhou^15^ showed that 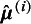 has the following asymptotic distribution at the within-study level: 
(3)

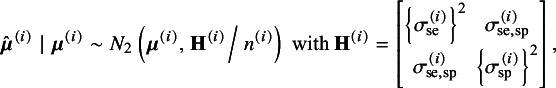

where 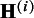 indicates the within-study asymptotic variance-covariance matrix. The detailed expression of 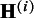 is presented in equation (S1) of the Supplementary Material. The parameter 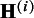 in model (3) can be replaced with its consistent estimator 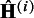 estimated by the Greenwood formula with median follow-up time, which follows the convention that the within-study variance–covariance matrix is known in meta-analysis. (See Section 3.2 in Hattori and Zhou^15^ for more details.) Combining models (2) and (3) induces the HZ model: 
(4)

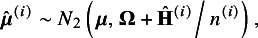

where 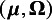 are the unknown parameters at time *t*, and they can be estimated by the maximum likelihood (ML) method; we denote their ML estimators as 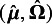 .

Based on the HZ model, 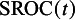 is derived by taking the conditional expectation of 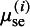 given 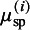 in model (2). Let *x* denote 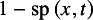 , 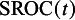 is defined by the following time-dependent function: 
(5)

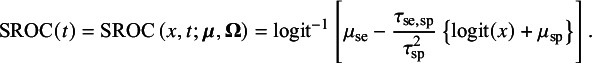

Accordingly, 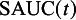 is defined by 
(6)

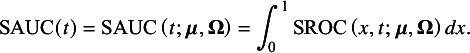




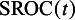
 and 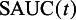 can be estimated by replacing the unknown parameters 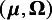 in (5) and (6).

## 4 Sensitivity analysis for reporting bias

### 4.1 Trivariate model incorporating the lnHR

In intervention studies, whether or not a study is published is influenced by the significance of the result, such as, the *P*-value of the *t*-statistic. Similarly, a prognosis study with time-to-event outcomes is more likely to be published when its log-rank test (or equivalently, the *t*-statistic of the lnHR without covariates) is significant, and vice versa. The phenomenon of selective publication causes *N* published studies to be biased sample from the *S* studies and may induce reporting bias in the estimates of 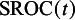 and 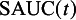 . To quantify reporting bias on the estimates, we aim to extend the methods of Copas^19^ and Zhou et al.^24^ and introduce the selection function of the log-rank test (equivalently, the *t*-statistic of the lnHR) to model the publication mechanism of prognosis studies; then, the inference of reporting bias is made based on the conditional likelihood of the HZ model given the published studies. However, the HZ model (4) does not involve the lnHR. Thus, before introducing the proposed method, we need to expand the HZ model (4) to correlate the time-dependent sensitivity and specificity with the lnHR for constructing the conditional likelihood in the later section.

To distinguish from some notations for the HZ model (4) in Section 3.2, we let 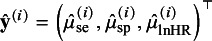 denote the consistent estimators of 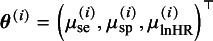 at time *t*, where 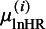 denotes the lnHR and 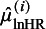 the empirical lnHR estimated by the Cox model from the published prognosis studies. At the between-study level, it is assumed that 
(7)

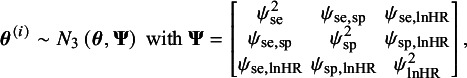

where 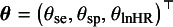 are the common means, and 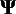 indicates the between-study variance-covariance matrix of 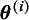 ; the diagonal elements are the corresponding variances of 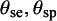 , and 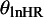 and the others are the covariances between each two of them.

At the within-study level, we can prove that 
(8)

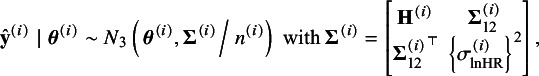

where 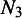 denotes the trivariate normal distribution, 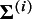 the within-study asymptotic variance–covariance matrix, 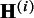 the variance–covariance matrix in model (3); let 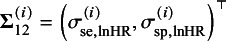 , where 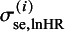 is the covariance between 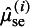 and 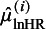 and 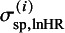 the covariance between 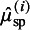 and 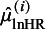 , and 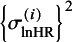 the variance of 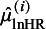 . The proof of the asymptotic distribution of 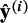 (8) is presented in Section S2 of the Supplementary Material. In (8), 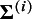 can be replaced by its consistent estimator 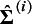 , according to the convention in meta-analysis that the within-study variance–covariance matrix is known. Combining models (8) and (7), we derives the marginal distribution of 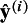 : 
(9)

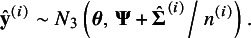



### 4.2 Selection functions on the significance of the lnHR

As aforementioned, we conjectured that the published *N* studies are subject to selective publication in that study with significant lnHR (or small *P*-value of the log-rank test) is more likely to be published. The selective publication process is modeled by the probability of a study being selected given its *t*-statistic of the lnHR, denoted by 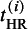 , as shown in the following selection function: 
(10)

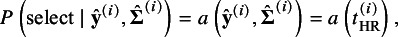

where the function 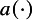 is a non-decreasing function of 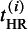 , that is, the *t*-statistic of the lnHR. To simplify the inference procedure, we, following Copas,^19^ employ the probit model to 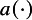 . Thus, equation (10) is defined by: 
(11)

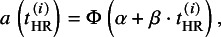

where 
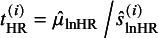
 , parameters 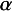 and 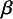 control the probability of selective publication, and 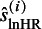 denotes the reported SE of the lnHR with 
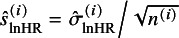
 . The monotonic property of the probit model links two cases of randomly selective publication: (1) when 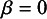 and suppose 
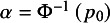
 , the probability of selective publication is independent of the *t*-statistic, and each study is randomly published from the population with selection probability 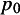 ; (2) when 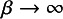 , each study is published with probability 1.

According to the definition of the probit model, the selection function (11) can be represented by 
(12)

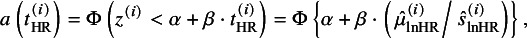

where 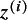 is the standard normal random variable independent of 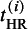 . Based on the trivariate model (9), the marginal distribution of 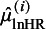 is 

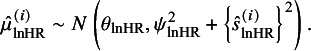

Then, the distribution of 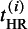 is derived by 

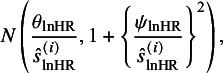

and the selection function 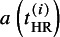 in equation (12) can be written into the following selection function: 
(13)

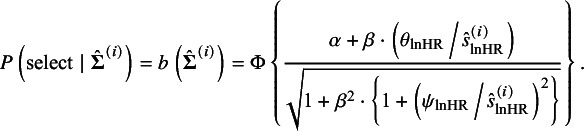



### 4.3 Likelihood based sensitivity analysis

We estimate the parameters in 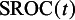 and 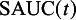 by maximizing the loglikelihood subject to a certain value of marginal selection probability, denoted by 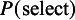 and defined by 
(14)

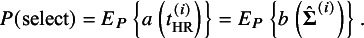

We regard 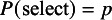 as the sensitivity parameter, implying the the expected proportion of the published from the population studies. With various values of *p*, the changes in the estimates of 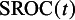 and 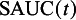 imply the impact of reporting bias on them. In this section, we derive the loglikelihood function conditional on the published studies at a fixed value of *p*.

We let 
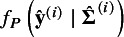
 denote the marginal distribution of 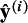 and 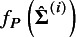 the distribution of 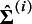 over the population studies. In meta-analysis without taking into account reporting bias, the empirical data 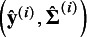 are regarded as random sample from the population studies with the joint distribution 
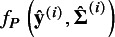
 . In the presence of selective publication, the published studies can be biased sample from the population; we let 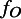 denote the distribution of the selectively published samples. Given a fixed *p*, the distribution of 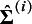 in the published studies is derived by 

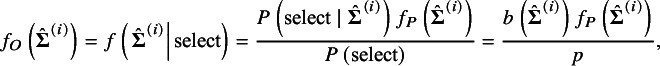

which gives 
(15)

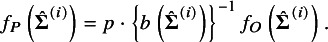

The joint distribution of the empirical data is then derived by 

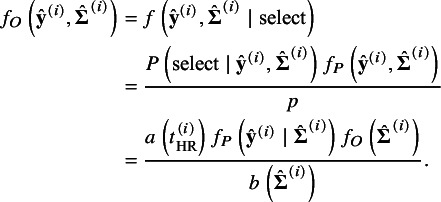

This joint distribution allows us to derive the loglikelihood of published studies, that is, 
(16)

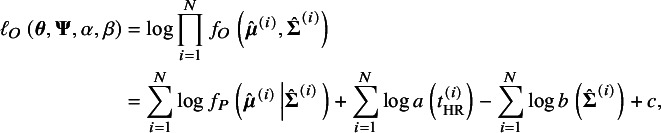

where the second and the third terms are used for correcting reporting bias, and 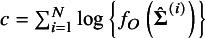 is constant. If a random selection of studies holds, then either 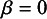 or 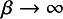 holds as well. Consequently, these two terms cancel each other, making the likelihood reduce to that without accounting for reporting bias.

Noting that by taking the integral of both sides in equation (15), we can derive 
(17)

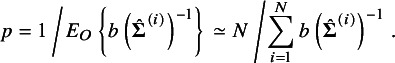

According to the definition of 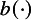 (13), equation (17) is monotonic with respect to 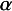 ; thus, the parameter 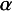 can be represented by the function of 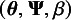 given a value of *p*. We denote this by 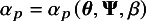 . By replacing the 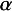 with 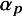 , we derive the conditional loglikelihood given the published studies from the loglikelihood (16): 
(18)

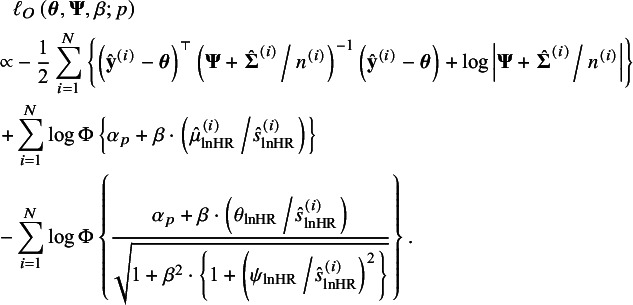

Differently from the loglikelihood (16), with 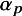 , this loglikelihood is constraint by the marginal selection probability. The parameters 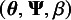 can be estimated by maximizing the conditional loglikelihood given a specified value of *p*. We denote these ML estimates as 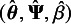 . With these MLEs, 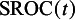 in equation (5) and 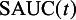 in equation (6) can be estimated accordingly, denoted by 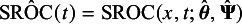 and 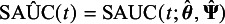 , respectively. The asymptotic normality of 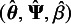 follows the general theory of the ML estimation under the assumptions that the number of studies and subjects are large. The asymptotic variance-covariance matrix of 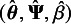 can be consistently estimated by the inverse of the empirical Fisher information following the ML theory. The two-tailed CI for the SAUC can be constructed by the delta method, and the detailed derivation is presented in Section S3 of the Supplementary Material.

In practice, the true value of *p* is unknown. As a sensitivity analysis for reporting bias, the value of *p* should be taken within the range 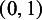 in general. It is recommended to specify a decreasing series for *p*, such as 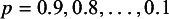 , to thoroughly examine the changes of 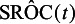 or 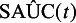 . Specifying a large value, such as 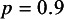 , implies that the published studies account for 90% of the population, and thereby small reporting bias will be corrected. In contrast, specifying a small value, such as 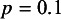 , indicates that the published studies represent 10% of the population, resulting in possibly substantial reporting bias to be corrected by the proposed method. If the range for the probability of publication can be reasonably conjectured, it is preferable to specify a decreasing series for *p* within that narrower range. In the following section, we illustrate the practical implementation of the proposed method.

## 5 Application

We revisited the meta-analysis of Ki67^8^ to evaluate the potential impact of reporting bias on 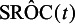 and 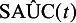 at 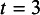 and 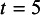 . As mentioned in Section 2, 38 studies reported the lnHRs for the overall survival outcome. Some potentially unpublished studies were detected in the lnHRs and the logit-transformed sensitivities by the funnel plots, as shown in Figure S1 in the Supplementary Material. When 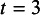 and 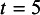 , only 23 and 21 studies reported the KM estimates, respectively. To evaluate the impact of reporting bias on 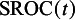 and 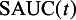 , we then adopted the proposed method. Considering that the number of population studies should be at least 38, the marginal selection probabilities 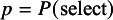 were estimated to be no greater than 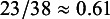 or 0.55, respectively. Thus, we considered implementing the proposed sensitivity analysis on the estimates of 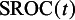 given 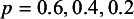 . As for 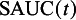 , we gave thorough sensitivity analysis given 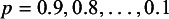 . In the estimations, we used the absolute value of the t-statistic, 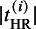 , in (11), indicating that the publication of studies was influenced by the two-tailed *P*-value of the lnHRs.

**Figure 3:**
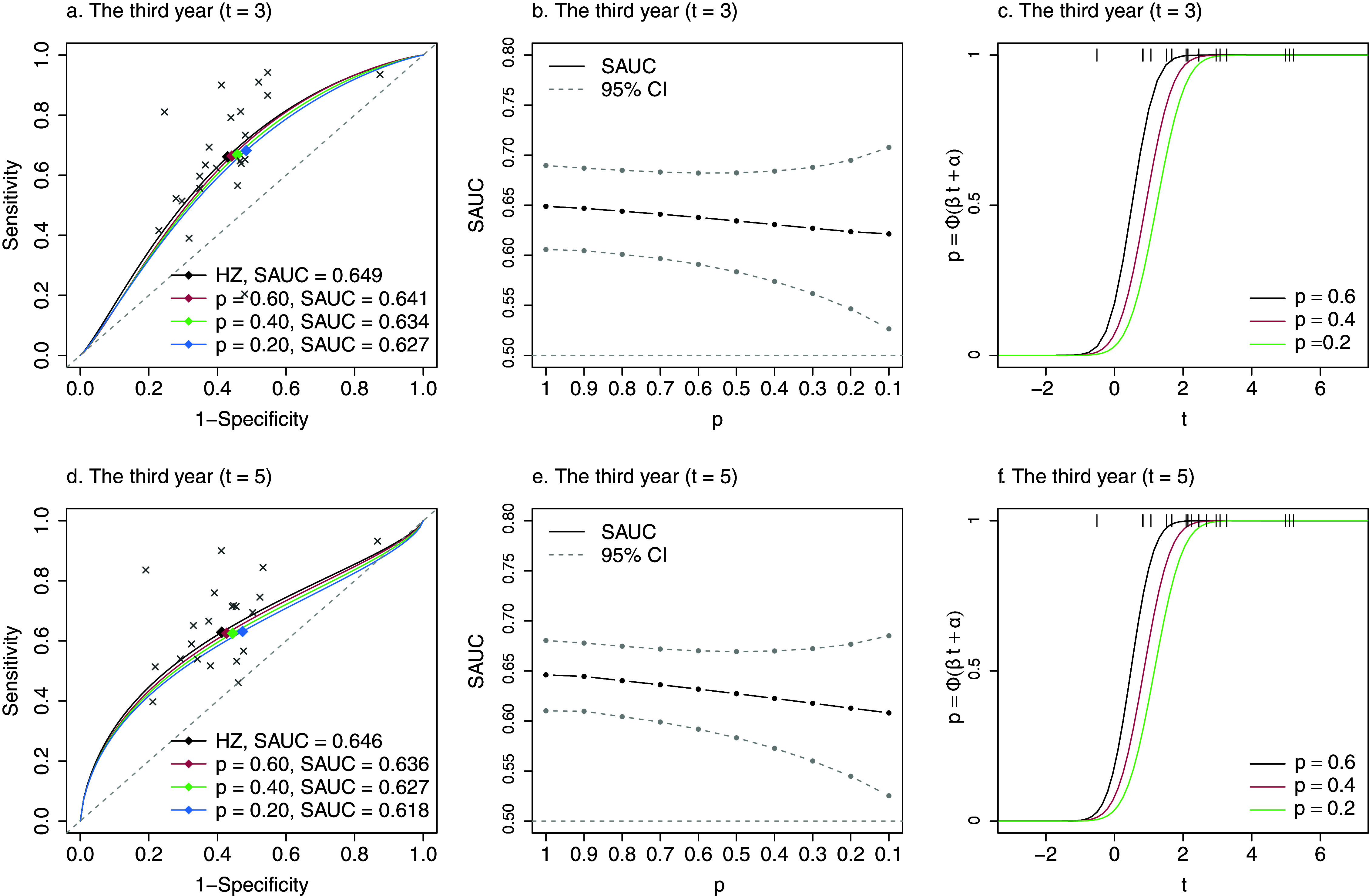
The SRÔC(t) and SAÛC(t), and the probit selection function at 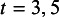 when 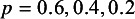 in Ki-67 example. In panels (a) and (d), the circle points are the empirical 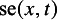 and 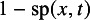 pairs from 23 prognosis studies; the diamond points are the estimated summary operating points, 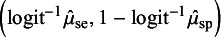 . Panel (b) and (e) show SAÛC(t) by the HZ model ( 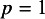 ) and the proposed method given 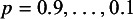 . In panels (c) and (f), the vertical lines at the top are the observed *t*-statistics from 23 prognosis studies.

We regarded estimates by the HZ model without taking into account reporting bias (when 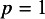 ) as the benchmark. The changes of 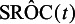 at 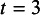 and 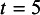 are shown in Figure 3a and 3d, respectively, with the estimates of corresponding parameters presented in Tables S4 and S5 in the Supplementary Material. Although there was a small impact of reporting bias on the 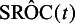 , the estimated summary operating points (the diamond points) were changed variously when 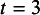 and 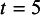 . At 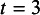 , with *p* decreasing to 0.2, the integrated sensitivity increased slightly, and the integrated specificity decreased (as 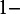 specificity increased). At 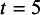 , the integrated sensitivities were almost unchanged, while the integrated specificity decreased when *p* decreased to 0.2. The different changes on the integrated sensitivity and specificity indicated that, in this example, the selective publication determined by the significance of the lnHRs had more impact on the estimates of specificity than sensitivity; thus, one should also be cautious about the inference of integrated specificity.

The HZ model estimated 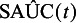 at 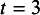 and 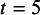 to be 0.649 (95% CI: 0.606, 0.690) and 0.646 (0.610, 0.680), respectively. Corresponding to the estimate of 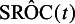 , when 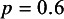 , that is, about 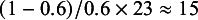 unpublished studies potentially existed, the estimated 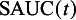 at 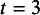 and 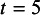 decreased to 0.638 (0.591, 0.682) and 0.632 (0.592, 0.677), respectively. In the worst case when 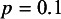 , 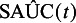 at 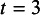 and 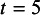 decreased to 0.621 (0.526, 0.708) and 0.608 (0.525, 0.685), respectively. The changes of 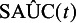 at 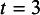 and 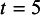 given 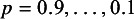 are shown in Figure 3b and 3e, respectively. Although 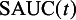 decreased with decreasing *p*, the estimates of 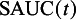 were still significantly different from 0.5. The estimated probit selection functions (equation 11) at 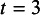 and 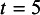 were presented in Figure 3c and 3f, respectively. The *t*-statistics of the published studies were shown as the vertical lines, and most studies had high probabilities of being selected. The detailed estimates of 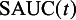 in Figure 3b and 3e were presented in Table S6 in the Supplementary Material.

Although the prognostic capacity of Ki67 was not high, 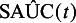 at 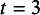 and 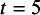 was estimated to be statistically significant by the HZ model. The sensitivity analysis supported the robustness of these estimates, indicating that 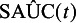 were affected by reporting bias to a small degree at the third ( 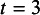 ) and the fifth ( 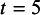 ) years. With the sensitivity analysis, one could draw the robust conclusion that the prognostic capacity of Ki67 antigen was not very high in patients with early breast cancer.

## 6 Simulation studies

Simulation studies were conducted to evaluate the performance of the proposed sensitivity analysis method. Following the data structure introduced in Section 3.1, we generated latent IPD in each prognosis study and used the observable data for meta-analysis. In each prognosis study *i*, we considered the following scenarios to generate the IPD.^15^ We considered one moderate size of total subjects with 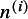 generated from the uniform distribution 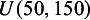 and one heterogeneous and large size with 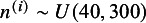 . The failure time of each subject 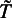 was generated from 
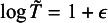
 , where 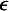 followed the standard normal distribution. The potential censored time 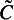 was generated from the exponential distribution with hazard rate 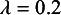 , denoted by 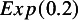 . Since the asymptotic variance–covariance matrices in equations (4) and (9) required the limiting variances of 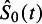 and 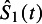 , the distribution of 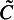 were estimated. We supposed 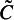 to be the exponential distribution. Following the method of Hattori and Zhou,^15^ we estimated 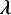 by using the empirical medians of the follow-up time. In simulation studies, we were interested in estimating 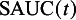 at 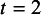 , denoted by SAUC(2). Different distributions of biomarkers were considered for subjects with failure time 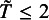 and 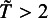 . Specifically, we considered five scenarios of the biomarker 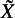 with normal distributed cutoff values having large or small variances; all the scenarios were summarized in Table 1.

With the IPD in study *i*, the sensitivity and specificity at 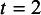 were estimated according to equation (1), and 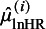 was estimated by the Cox model on the biomarker 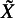 . The asymptotic variance–covariance matrix in the trivariate model (7) was derived according to equations (S1), (S9), and (S10) in the Supplementary Material.

For meta-analysis, we considered 70% and 50% of studies were published from the population, that is, the marginal selection probability 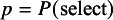 was set as 0.7 or 0.5, respectively. We considered small, medium, and large sizes of published studies, that is, about 25, 50, and 140 or 150 studies to be published. In each selective publication process, we generated *S* population studies and then selected 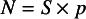 studies according to the probit selection function (11). In the true probit selection function, 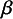 was set to be 5, and the values of 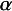 at 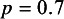 or 0.5 were calculated according to the definition of 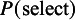 in equation (14). The corresponding values of 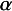 were presented in Table S7 in the Supplementary Material; the detailed data-generating process was presented in Section S5.1 of the Supplementary Material. The publication process was repeated 1,000 times in each scenario.

In the absence of reporting bias, SAUC(2) was estimated by maximizing the likelihood of the HZ model based on *S* population studies, where results were denoted by BNM 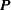 ; SAUC(2) with reporting bias were estimated by the HZ model with *N* published studies, and results were denoted by BNM 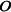 . The impact of reporting bias on SAUC(2) was indicated by the differences between the estimates of BNM 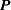 and BNM 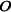 . The proposed method was applied to the data of *N* published studies and its results were denoted by Proposed. The difference between the estimates of BNM 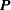 and proposed implied the bias of the proposed method. The estimated 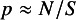 was used as a constraint to optimize the parameters. In practice, estimating *p* was infeasible; in simulation studies, *p* could be estimated and was used as the specified value for evaluating the performance of the proposed method.

**Table 2:** Summary of estimates of SAUC(2) by the HZ model and the proposed method when censoring distribution is correctly specified and 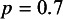

Patients	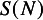	B	BNM 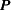	BNM 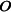	Proposed	CR (%)	RB	Bias
50–150	35 (25)	1	70.82 (69.67, 72.07)	72.47 (70.77, 74.25)	72.17 (70.28, 74.17)	67.3	1.65	1.35
		2	76.88 (75.76, 77.99)	78.44 (77.17, 79.98)	78.36 (76.60, 80.10)	68.7	1.57	1.48
		3	57.86 (56.95, 58.66)	60.35 (59.35, 61.21)	58.95 (57.87, 59.98)	67.8	2.49	1.09
		4	65.13 (63.93, 66.38)	67.54 (66.49, 68.78)	66.48 (65.37, 67.72)	68.1	2.41	1.35
		5	65.06 (64.10, 66.07)	67.55 (66.46, 68.56)	66.18 (65.02, 67.45)	63.6	2.50	1.12
		6	66.95 (66.03, 67.85)	69.53 (68.64, 70.44)	68.18 (67.06, 69.26)	65.2	2.58	1.23
	70 (49)	1	70.84 (70.01, 71.60)	72.46 (71.34, 73.70)	72.15 (70.54, 73.67)	85	1.63	1.32
		2	76.78 (76.00, 77.66)	78.44 (77.42, 79.47)	78.33 (77.06, 79.50)	80.7	1.66	1.55
		3	57.79 (57.17, 58.37)	60.33 (59.77, 61.00)	58.57 (57.81, 59.40)	82.8	2.54	0.78
		4	65.15 (64.27, 65.93)	67.48 (66.56, 68.31)	66.20 (65.27, 67.12)	81.1	2.33	1.05
		5	65.10 (64.40, 65.79)	67.49 (66.66, 68.20)	66.13 (65.23, 66.96)	76.3	2.39	1.03
		6	67.04 (66.37, 67.66)	69.65 (69.02, 70.29)	67.96 (67.02, 68.76)	78.4	2.61	0.92
	200 (140)	1	70.63 (70.11, 71.17)	72.18 (71.45, 72.84)	71.57 (70.70, 72.58)	89.9	1.55	0.94
		2	76.84 (76.36, 77.34)	78.38 (77.62, 79.15)	78.20 (77.38, 79.06)	81.1	1.54	1.36
		3	57.80 (57.44, 58.14)	60.34 (59.98, 60.75)	58.51 (58.06, 58.96)	90.1	2.55	0.72
		4	65.02 (64.45, 65.52)	67.45 (66.88, 67.96)	66.10 (65.53, 66.66)	85.6	2.43	1.08
		5	65.03 (64.58, 65.46)	67.42 (66.91, 67.93)	65.97 (65.49, 66.54)	81.8	2.38	0.94
		6	67.05 (66.65, 67.46)	69.62 (69.28, 70.05)	67.74 (67.26, 68.26)	86.4	2.57	0.69
40–300	35 (25)	1	71.25 (70.31, 72.28)	72.07 (70.76, 73.42)	71.72 (70.14, 73.37)	66.6	0.83	0.47
		2	77.12 (76.31, 77.97)	77.94 (76.75, 79.10)	77.58 (76.28, 78.95)	62.3	0.82	0.46
		3	58.07 (57.34, 58.73)	59.82 (59.16, 60.57)	58.82 (57.90, 59.67)	57.4	1.75	0.76
		4	65.50 (64.56, 66.38)	67.07 (66.11, 67.91)	66.41 (65.37, 67.45)	59.8	1.57	0.91
		5	65.65 (64.86, 66.37)	67.27 (66.46, 68.04)	66.54 (65.45, 67.60)	55.7	1.62	0.89
		6	67.30 (66.55, 68.00)	69.02 (68.24, 69.74)	68.07 (66.95, 69.01)	57.2	1.72	0.77
	70 (49)	1	71.24 (70.59, 71.97)	72.02 (71.11, 73.06)	71.73 (70.65, 72.91)	84.6	0.79	0.49
		2	77.13 (76.57, 77.77)	77.93 (77.09, 78.78)	77.45 (76.62, 78.52)	75.8	0.81	0.32
		3	58.00 (57.55, 58.45)	59.87 (59.32, 60.33)	58.62 (57.97, 59.20)	70.9	1.87	0.62
		4	65.51 (64.84, 66.19)	67.08 (66.42, 67.72)	66.53 (65.72, 67.19)	72.9	1.57	1.02
		5	65.52 (65.01, 66.06)	67.10 (66.54, 67.65)	66.27 (65.54, 66.97)	69.8	1.58	0.74
		6	67.37 (66.91, 67.90)	69.13 (68.58, 69.61)	68.06 (67.29, 68.71)	75.5	1.76	0.69
	200 (140)	1	71.16 (70.76, 71.57)	71.84 (71.27, 72.45)	71.52 (70.92, 72.26)	89.9	0.68	0.36
		2	77.18 (76.85, 77.57)	78.05 (77.53, 78.62)	77.74 (77.14, 78.32)	73.4	0.88	0.56
		3	57.96 (57.71, 58.27)	59.81 (59.54, 60.11)	58.48 (58.17, 58.83)	75.1	1.85	0.52
		4	65.51 (65.11, 65.89)	67.07 (66.67, 67.46)	66.32 (65.86, 66.79)	76.2	1.57	0.81
		5	65.52 (65.18, 65.84)	67.08 (66.75, 67.46)	66.19 (65.76, 66.63)	69.7	1.57	0.67
		6	67.38 (67.06, 67.67)	69.06 (68.77, 69.37)	67.92 (67.45, 68.30)	81.5	1.68	0.54

*Note*: B denotes the scenarios of biomarker coresponding to Table 2; CR shows convergence rate of the proposed method; estimates are summarized by median (first quantile, third quantile); RB denotes reporting bias; Bias denotes bias of the proposed method.

**Table 3:** Summary of estimates of SAUC(2) by the HZ model and the proposed method when censoring distribution is correctly specified and 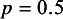

Patients	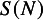	B	BNM 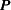	BNM 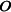	Proposed	CR (%)	RB	center
50–150	50 (25)	1	70.79 (69.75, 71.87)	73.18 (71.46, 74.98)	71.99 (69.94, 74.14)	69.9	2.39	1.20
		2	76.86 (75.90, 77.79)	78.73 (77.18, 80.13)	78.00 (75.73, 79.67)	66.8	1.87	1.14
		3	57.83 (57.09, 58.54)	61.85 (60.92, 62.71)	58.70 (57.45, 59.76)	70.4	4.02	0.87
		4	65.14 (64.19, 66.12)	68.90 (67.70, 69.98)	66.26 (65.01, 67.51)	68.5	3.76	1.12
		5	65.09 (64.26, 65.89)	68.88 (67.89, 69.88)	65.98 (64.73, 67.25)	66.1	3.79	0.89
		6	66.96 (66.18, 67.67)	71.01 (70.10, 71.91)	67.91 (66.59, 69.04)	65.4	4.06	0.95
	100 (50)	1	70.71 (70.07, 71.35)	73.12 (71.82, 74.23)	71.61 (70.07, 73.40)	86	2.40	0.89
		2	76.80 (76.11, 77.52)	78.59 (77.52, 79.56)	77.74 (76.38, 78.97)	77.1	1.79	0.94
		3	57.81 (57.29, 58.29)	61.84 (61.20, 62.42)	58.36 (57.61, 59.14)	85.9	4.04	0.56
		4	65.12 (64.41, 65.81)	68.80 (68.01, 69.60)	66.08 (65.10, 66.94)	81.3	3.69	0.96
		5	65.08 (64.54, 65.63)	68.86 (68.09, 69.57)	65.92 (64.97, 66.70)	76.9	3.78	0.85
		6	67.04 (66.48, 67.60)	71.15 (70.54, 71.74)	67.69 (66.82, 68.53)	80.1	4.11	0.65
	300 (150)	1	70.61 (70.19, 71.01)	72.85 (72.19, 73.58)	71.40 (70.51, 72.49)	90.4	2.24	0.79
		2	76.87 (76.45, 77.26)	78.45 (77.75, 79.09)	77.61 (76.87, 78.44)	75.7	1.58	0.75
		3	57.80 (57.53, 58.10)	61.88 (61.52, 62.26)	58.38 (57.92, 58.82)	88.5	4.08	0.59
		4	65.02 (64.55, 65.43)	68.78 (68.16, 69.23)	65.95 (65.34, 66.48)	83.1	3.76	0.94
		5	65.00 (64.68, 65.37)	68.70 (68.20, 69.17)	65.66 (65.17, 66.26)	76	3.69	0.66
		6	67.04 (66.72, 67.40)	71.16 (70.79, 71.52)	67.64 (67.06, 68.10)	84.5	4.12	0.59
40–300	50 (25)	1	71.24 (70.51, 72.06)	72.39 (70.94, 73.77)	71.31 (69.62, 73.05)	70.1	1.15	0.07
		2	77.11 (76.43, 77.87)	77.88 (76.74, 78.95)	77.11 (75.52, 78.51)	63.4	0.77	−0.00
		3	58.04 (57.46, 58.60)	60.95 (60.32, 61.60)	58.72 (57.82, 59.56)	65.1	2.91	0.68
		4	65.43 (64.75, 66.21)	67.93 (67.09, 68.75)	66.39 (65.21, 67.36)	62.9	2.49	0.96
		5	65.60 (65.01, 66.21)	68.19 (67.37, 68.95)	66.49 (65.33, 67.47)	58.5	2.59	0.89
		6	67.31 (66.66, 67.90)	70.06 (69.36, 70.76)	67.89 (66.63, 68.84)	61.1	2.75	0.58
	100 (50)	1	71.25 (70.68, 71.83)	72.28 (71.25, 73.35)	71.37 (70.19, 72.58)	85.4	1.03	0.13
		2	77.13 (76.63, 77.66)	77.76 (76.92, 78.54)	76.89 (75.91, 77.84)	72.8	0.62	−0.24
		3	57.97 (57.59, 58.36)	60.98 (60.49, 61.44)	58.57 (57.86, 59.17)	75.9	3.00	0.60
		4	65.51 (64.98, 66.02)	67.95 (67.39, 68.56)	66.32 (65.58, 67.05)	74.6	2.44	0.80
		5	65.53 (65.11, 65.95)	68.03 (67.53, 68.54)	66.21 (65.56, 66.90)	70.3	2.50	0.68
		6	67.41 (66.99, 67.87)	70.15 (69.62, 70.61)	67.87 (67.07, 68.60)	76.7	2.74	0.46
	300 (151)	1	71.14 (70.81, 71.48)	72.05 (71.48, 72.58)	71.21 (70.47, 71.87)	86.1	0.90	0.07
		2	77.19 (76.92, 77.47)	77.83 (77.31, 78.30)	77.14 (76.55, 77.74)	65.3	0.64	−0.05
		3	57.99 (57.74, 58.19)	60.92 (60.66, 61.18)	58.45 (58.11, 58.79)	71.3	2.93	0.46
		4	65.50 (65.22, 65.80)	67.99 (67.64, 68.38)	66.27 (65.79, 66.68)	73.9	2.49	0.77
		5	65.51 (65.26, 65.78)	68.06 (67.73, 68.36)	66.12 (65.72, 66.59)	63.4	2.55	0.61
		6	67.39 (67.12, 67.63)	70.12 (69.83, 70.42)	67.73 (67.30, 68.19)	78.3	2.73	0.34

*Note*: B denotes the scenarios of biomarker coresponding to Table 2; CR shows convergence rate of the proposed method; estimates are summarized by median (first quantile, third quantile); RB denotes reporting bias; Bias denotes bias of the proposed method.

All statistical computing was conducted by R (R Development Core Team, Version 4.1.3). The ML estimations were optimized by the Newton–Raphson method and conducted by R function nlminb(). The lnHR was estimated by the Cox model conducted by the R package survival.^28^ The details of optimization were presented in Section S5.2 of the Supplementary Material.

We considered the situation when the censoring distribution was correctly fitted by the exponential distribution and estimated the hazard rate using the medians of the simulated follow-up time. The medians with the first and the third quartiles of the estimated SAUC(2) in all the scenarios when 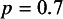 and 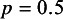 were summarized in Tables 2 and 3, respectively. With the setting of a smaller 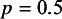 , reporting bias, as shown in RB, increased in Table 3 comparing to the case of 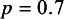 in Table 2. The magnitude of reporting bias was observed to be greater in Biomarker Scenarios 3–6, indicating that the bias increased with increasing variances of biomarkers. When the censoring distribution was correctly fitted in the estimation, the proposed method reduced reporting bias in all the scenarios, especially when the number of studies (*N*) was large. When the number of patients was larger, the bias of the proposed method as well as reporting bias decreased. In addition, we summarized the corresponding estimates of time-dependent sensitivity and specificity; the results were presented in Tables S8–S11 in the Supplementary Material. We observed larger reporting bias on the estimates of sensitivity than specificity for Biomarker Scenarios 1–5, where smaller variances were set for biomarkers given failure time 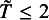 . Conversely, in scenario 6, where larger variances were set for biomarkers given failure time 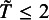 , larger reporting bias was found on the estimates of specificity. We observed that the magnitude of reporting bias varied in estimates of sensitivity or specificity due to differences in data, and regardless of the scenarios, the proposed methods successfully reduced biases on both sensitivity and specificity.

The convergence rates (CRs) of the proposed method were also presented; the CR was calculated by the proportion of successfully obtaining the converged estimates among 1,000 repetitions. Since the proposed method was based on the trivariate normal model and contained 11 parameter, when the number of published studies was small, the performance of the proposed method showed comparatively low CR. When the number of patients were large, reporting bias was observed to be decreased, and the proposed method could still reduce reporting bias in the estimates.

We also considered the situations when censoring distribution was misspecified in the proposed method given 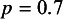 . We generated the true censoring distribution by the uniform distribution 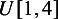 and lognormal distribution 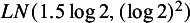 . In the proposed method, we still used the exponential distribution to estimate the censoring distribution incorrectly. Under the misspecification of the censoring distribution, we evaluated the performance of the proposed methods. The estimates were summarized in Tables S12 and S13 in the Supplementary Material. Even with the misspecification of the censoring distribution, the proposed method could reduce reporting bias. The results were in agreement with those when the censoring distribution was correctly fitted; however, the misspecification of the censoring distribution caused the CRs of the estimation reduced, especially when the number of studies was small or the number of patients was large.

Overall, simulation studies showed that the proposed method could reduce reporting bias. However, since the proposed methods needed to estimate ten unknown parameters, the unconverged estimates were obtained by using nlminb(), especially when the number of published studies was small or the underlying censoring distribution was misfitted. With enough number of published studies, the simulation studies showed that the proposed method could obtain CR around 70% or more when 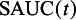 were better than moderate. In practice, we suggest trying different plausible initial values such as some values that are speculated to be close to the true values of some parameters.

## 7 Discussion

Reporting bias is widely recognized as a major issue in various meta-analyses and may lead to over-optimistic conclusions. Thus, addressing reporting bias is a crucial part of meta-analysis, enhancing the reliability of meta-analytical results. In recent decades, meta-analysis of prognosis studies has been gaining increasing interest in medical research. Riley et al.^29^ discussed the importance of meta-analysis of prognosis studies and summarized several challenges specific for it. The choice of cutoff value is one of the issues. With a continuous biomarker, different choice of cutoff values would lead to great heterogeneity in meta-analysis and difficulty in interpreting the results.^29^ However, in practice, almost all the clinical reports of meta-analysis of prognostic studies simply applied the standard meta-analysis technique to aggregate the outcomes, such as the HRs, of individual studies, ignoring the heterogeneous cutoff values. The underdevelopment of relevant statistical methods may account for such phenomena. The time-dependent SROC curve has been proposed to show the summary prognostic capacity independent of cutoff values.^14^ ^,^ ^15^ Considering the wide acceptance of the SROC method in diagnostic meta-analysis, these 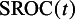 -based methodologies should be emphasized and applied more in practice. Meanwhile, there is a growing need to develop corresponding methods addressing reporting bias in these meta-analytical methodologies. In general, our proposed method is expected to enhance the utility of 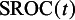 method in evaluating prognostic capacity of biomarkers and moreover reduce the possible reporting bias on the meta-analytical results. Our method is developed as a subordinate analysis to the bivariate normal model by Hattori and Zhou.^15^ Their model is much simpler in the inference than the method of Combesecure et al.^14^ and the bivariate binomial model of Hattori and Zhou.^15^ Our proposal provides a mean for researchers to draw more reliable conclusions about prognostic capacity from their meta-analysis.

While many methods have been proposed to deal with reporting bias on the aggregated HRs, to the best of our knowledge, no methods exist for reporting bias on 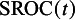 -based results. Our proposal is the first tool to evaluate the robustness of 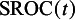 -based results against reporting bias. However, several limitations should be acknowledged. The proposed method relies on the trivariate normal distribution, which involves numerous parameters. Nonconvergence issues may occur in some small meta-analyses; however, simulation studies indicated that meta-analysis with at least 25 studies achieved an acceptable convergence rate. Enhancing the estimation strategy remains an area for future improvement. On the other hand, our proposal employs the parametric probit selection function, which is theoretically sound^30^ for modeling selective publication process of prognostic studies and allows the derivation of a closed-form likelihood; however, the suitability of the probit model cannot be checked from the observed data. Recently, Zhou et al.^31^ proposed non-parametric worst-case bounds to deal with reporting bias on the SROC/SAUC estimations in diagnostic meta-analysis. Their method utilized a class of nonparametric selection functions under the assumption that larger studies with possibly significant sensitivity or specificity are more likely to be published. The non-parametric worst-case bounds method provides an alternative framework addressing reporting bias and could potentially be adapted to the HZ model for prognostic meta-analysis in the future.

Our proposal put forward an idea for modeling reporting bias in prognosis studies with time-to-event outcomes and may help the development of new statistical methods dealing with reporting bias on various estimates in meta-analysis of prognosis studies. The bivariate binomial model of Hattori and Zhou^15^ is based on the exact likelihood to model the numbers of survived and failed subjects and may outperform the HZ model when the number of studies is small. Hattori and Zhou^32^ developed a meta-analytic version of the concordance index for the time-to-event outcome. Our proposal is expected to be extended to address reporting bias in these meta-analytical models.

## Supporting information

Zhou et al. supplementary material Zhou et al. supplementary material

## Data Availability

R codes together with a sample application data set are available at https://github.com/meta2020/progmetasa-r.

## Supplementary material

The supplementary material for this article can be found at https://doi.org/10.1017/rsm.2025.14.
