# Supplementary material for: Sensitivity analysis for reporting bias on the time-dependent summary receiver operating characteristics curve in meta-analysis of prognosis studies with time-to-event outcomes
Source: Res Synth Methods. 2025 Mar 21;16(3):528–49. doi: 10.1017/rsm.2025.14 (PMC12527534; doi:10.1017/rsm.2025.14)
Supplement: Zhou et al. supplementary material [file S1759287925000146sup001.pdf]

Received: Added at production | Revised: Added at production | Accepted: Added at production

DOI: xxx/xxxx

SUPPLEMENTARY MATERIAL

Supplementary Material for “Sensitivity analysis for reporting bias on the time-dependent summary receiver operating characteristics curve in meta-analysis of prognosis studies with time-to-event outcomes”

Yi Zhou[1,2,3] | Ao Huang[4] | Satoshi Hattori*[2,5]

[1]Beijing International Center for Mathematical Research, Peking University, Beijing, China
[2]Department of Biomedical Statistics, Graduate School of Medicine, Osaka University, Osaka, Japan
[3]Graduate School of Human Development and Environment, Kobe University, Kobe, Japan
[4]Department of Medical Statistics, University Medical Center Göttingen, Göttingen, Germany
[5]Integrated Frontier Research for Medical Science Division, Institute for Open and Transdisciplinary Research Initiatives (OTRI), Osaka University, Osaka, Japan

This is the supplementary material for “Sensitivity analysis for reporting bias on the time-dependent summary receiver operating characteristics curve in meta-analysis of prognosis studies with time-to-event outcomes” by Yi Zhou, Ao Huang, and Satoshi Hattori.

S1 | DATA STRUCTURE OF META-ANALYSIS OF KI67

As mentioned in Section 2 of the main text, we give an example of the observable data for meta-analysis. The example showed data of the first several studies in meta-analysis of Ki67. The full data are accessible at https://github.com/meta2020/prgmetasa-r.

Table S1 presents the extracted log-transformed hazard ratios (lnHRs) between high versus low expression groups. The standard errors (SEs) of the lnHR can be derived from the 95% confidence intervals (CIs), that is, $\hat{s}^{(i)}_{\text{lnHR}}$ = (upper limit − lower limit)/3.92.

Table S2 presents the extracted medians of follow-up time and the corresponding KM estimates in high and low expression groups.

Table S3 presents the extracted number of subjects in high and low expression groups and the Kaplan-Meier (KM) estimates at different time points $t_0 < t_1 < \cdots < t_k < \cdots < t_K$.

TABLE S1 The extracted data of hazard ratio (HR) and the confidence intervals

Author	Study (i)	Cutoff value ($v^{(i)}$)	HR ($\exp\{\hat{\mu}^{(i)}_{\ln HR}\}$)	95% CI	Z
Bevilacqua	1	0.1	2.75	(1.02, 7.39)	1
Bos	2	0.1	2.47	(1.08, 5.65)	0
Brown	3	0.05	1.19	(0.79, 1.8)	0
Caly	4	0.32	1.95	(0.92, 4.14)	1
Domagala_no-	5	0.1	3.04	(1.03, 8.99)	1
...	...	...	...	...	...

$Z = 1$ if the study reports the KM curves and $Z = 0$ otherwise.

TABLE S2 The extracted medians of follow-up time and KM estimates

Author	Study (i)	Median follow-up month ($t_f^{(i)}$)	$\hat{S}_1(t_f)^{(i)}$	$\hat{S}_0(t_f)^{(i)}$
Bevilacqua	1	74	0.79	0.84
Erdern	7	72.5	0.6	0.97
Fresno	8	75	0.75	0.9
Goodson	11	61.2	0.75	0.9
Jansen	15	128	0.43	0.6
...	...	...	...	...

TABLE S3 The extracted number of subjects and KM estimates at various time points

Author	Study (i)	Months (t_k)	$n_1^{(i)}$	$n_0^{(i)}$	$\hat{S}_1(t)^{(i)}$	$\hat{S}_0(t)^{(i)}$
Bevilacqua	1	6	94	13	1	0.99
Bevilacqua	1	12	94	13	1	0.97
Bevilacqua	1	18	94	13	1	0.97
Bevilacqua	1	24	94	13	0.97	0.965
Bevilacqua	1	30	94	13	0.92	0.965
Bevilacqua	1	36	94	13	0.92	0.96
Bevilacqua	1	42	94	13	0.87	0.94
Bevilacqua	1	48	94	13	0.85	0.93
Bevilacqua	1	54	94	13	0.82	0.91
Bevilacqua	1	60	94	13	0.81	0.9
Bevilacqua	1	66	94	13	0.79	0.87
Bevilacqua	1	72	94	13	0.79	0.85
Caly	4	6	122	122	1	1
Caly	4	12	122	122	0.98	0.98
Caly	4	18	122	122	0.95	0.97
Caly	4	24	122	122	0.92	0.95
Caly	4	30	122	122	0.88	0.93
Caly	4	36	122	122	0.85	0.92
Caly	4	42	122	122	0.82	0.9
Caly	4	48	122	122	0.78	0.89
Caly	4	54	122	122	0.74	0.88
Caly	4	60	122	122	0.7	0.88
Caly	4	66	122	122	0.7	0.85
Caly	4	72	122	122	0.63	0.82
Domagala_no-	5	6	66	45	1	1
Domagala_no-	5	12	66	45	1	1
Domagala_no-	5	18	66	45	0.92	1
Domagala_no-	5	24	66	45	0.92	1
Domagala_no-	5	30	66	45	0.8	0.98
Domagala_no-	5	36	66	45	0.78	0.95
Domagala_no-	5	42	66	45	0.65	0.93
Domagala_no-	5	48	66	45	0.65	0.9
Domagala_no-	5	54	66	45	0.63	0.88
Domagala_no-	5	60	66	45	0.6	0.8
...	...	...	...	...	...	...

S2 | THE ASYMPTOTIC DISTRIBUTION OF $\hat{y}^{(i)}$ CONDITIONAL ON $\theta^{(i)}$

As mentioned in Section 4.1 of the main text, we prove that $\hat{\boldsymbol{y}}^{(i)} = \left(\hat{\mu}_{\mathrm{se}}^{(i)}, \hat{\mu}_{\mathrm{sp}}^{(i)}, \hat{\mu}_{\mathrm{lnHR}}^{(i)}\right)^{\top}$ has the following asymptotic distribution, as mentioned in equation (8) of the main text:

$$\sqrt{n^{(i)}}\begin{pmatrix}\hat{\mu}_{\mathrm{se}}^{(i)}-\mu_{\mathrm{se}}^{(i)}\\ \hat{\mu}_{\mathrm{sp}}^{(i)}-\mu_{\mathrm{sp}}^{(i)}\\ \hat{\mu}_{\mathrm{lnHR}}^{(i)}-\mu_{\mathrm{lnHR}}^{(i)}\end{pmatrix}\left|\begin{pmatrix}\mu_{\mathrm{se}}^{(i)}\\ \mu_{\mathrm{sp}}^{(i)}\\ \mu_{\mathrm{lnHR}}^{(i)}\end{pmatrix}\right. \xrightarrow{D} N_3\left(\mathbf{0},\boldsymbol{\Sigma}^{(i)}\right),$$

with

$$\boldsymbol{\Sigma}^{(i)} = \begin{bmatrix}\left\{\sigma_{\mathrm{se}}^{(i)}\right\}^2 & \sigma_{\mathrm{se,sp}}^{(i)} & \sigma_{\mathrm{se,lnHR}}^{(i)}\\ \sigma_{\mathrm{se,sp}}^{(i)} & \left\{\sigma_{\mathrm{sp}}^{(i)}\right\}^2 & \sigma_{\mathrm{sp,lnHR}}^{(i)}\\ \sigma_{\mathrm{se,lnHR}}^{(i)} & \sigma_{\mathrm{sp,lnHR}}^{(i)} & \left\{\sigma_{\mathrm{lnHR}}^{(i)}\right\}^2\end{bmatrix}.$$

By replacing the theoretical quantities in $\boldsymbol{\Sigma}^{(i)}$ with the corresponding consistent estimators, we can obtain the consistent estimator of $\boldsymbol{\Sigma}^{(i)}$, denoted by $\hat{\boldsymbol{\Sigma}}^{(i)}$.

S2.1 | $\mathbf{H}^{(i)}$: the asymptotic variance-covariance matrix of $\left(\hat{\mu}_{\mathrm{se}}^{(i)}, \hat{\mu}_{\mathrm{sp}}^{(i)}\right)^{\top}$

Hattori and Zhou gave the proof of the asymptotic variance-covariance matrix of $\left(\hat{\mu}_{\mathrm{se}}^{(i)}, \hat{\mu}_{\mathrm{sp}}^{(i)}\right)^{\top}$ in their Appendices A and B. Following their notations, we define $x = S_1^{(i)}(t)$, $y = S_0^{(i)}(t)$; $z = q_1^{(i)} = P\left(\tilde{X}_j > v^{(i)}\right)$, and $w = q_0^{(i)} = P\left(\tilde{X}_j \le v^{(i)}\right)$. Define $\mu_{\mathrm{se}}^{(i)} = \mathrm{logit}\left\{\mathrm{se}(v^{(i)},t)\right\} = g_{\mathrm{se}}\left\{S_1^{(i)}(t), S_0^{(i)}(t), q_1^{(i)}, q_0^{(i)}\right\} = g_{\mathrm{se}}^{(i)}(x,y,z,w)$ and, in the same way, $\mu_{\mathrm{sp}}^{(i)} = g_{\mathrm{sp}}^{(i)}(x,y,z,w)$. It has been proved that

$$\sqrt{n^{(i)}}\begin{pmatrix}\hat{\mu}_{\mathrm{se}}^{(i)}-\mu_{\mathrm{se}}^{(i)}\\ \hat{\mu}_{\mathrm{sp}}^{(i)}-\mu_{\mathrm{sp}}^{(i)}\end{pmatrix}\left|\begin{pmatrix}\mu_{\mathrm{se}}^{(i)}\\ \mu_{\mathrm{sp}}^{(i)}\end{pmatrix}\right. \xrightarrow{D} N_2\left(\mathbf{0},\mathbf{H}^{(i)}\right), \text{ with } \mathbf{H}^{(i)} = \begin{bmatrix}\left\{\sigma_{\mathrm{se}}^{(i)}\right\}^2 & \sigma_{\mathrm{se,sp}}^{(i)}\\ \sigma_{\mathrm{se,sp}}^{(i)} & \left\{\sigma_{\mathrm{sp}}^{(i)}\right\}^2\end{bmatrix},$$

and

$$\begin{aligned}\left\{\sigma_{\mathrm{se}}^{(i)}\right\}^2 &= \left\{\dot{g}_{\mathrm{se},x}^{(i)}\right\}^2\left(1/q_1^{(i)}\right)\left\{\sigma_1^{(i)}(t)\right\}^2 + \left\{\dot{g}_{\mathrm{se},y}^{(i)}\right\}^2\left(1/q_0^{(i)}\right)\left\{\sigma_0^{(i)}(t)\right\}^2\\ &\quad+\left\{\dot{g}_{\mathrm{se},z}^{(i)}-\dot{g}_{\mathrm{se},w}^{(i)}\right\}^2 q_1^{(i)}q_0^{(i)},\\ \left\{\sigma_{\mathrm{sp}}^{(i)}\right\}^2 &= \left\{\dot{g}_{\mathrm{sp},x}^{(i)}\right\}^2\left(1/q_1^{(i)}\right)\left\{\sigma_1^{(i)}(t)\right\}^2 + \left\{\dot{g}_{\mathrm{sp},y}^{(i)}\right\}^2\left(1/q_0^{(i)}\right)\left\{\sigma_0^{(i)}(t)\right\}^2\\ &\quad+\left\{\dot{g}_{\mathrm{sp},z}^{(i)}-\dot{g}_{\mathrm{sp},w}^{(i)}\right\}^2 q_1^{(i)}q_0^{(i)},\\ \sigma_{\mathrm{se,sp}}^{(i)} &= \dot{g}_{\mathrm{se},x}^{(i)}\dot{g}_{\mathrm{sp},x}^{(i)}\left(1/q_1^{(i)}\right)\left\{\sigma_1^{(i)}(t)\right\}^2 + \dot{g}_{\mathrm{se},y}^{(i)}\dot{g}_{\mathrm{sp},y}^{(i)}\left(1/q_0^{(i)}\right)\left\{\sigma_0^{(i)}(t)\right\}^2\\ &\quad+\left(\dot{g}_{\mathrm{se},z}^{(i)}-\dot{g}_{\mathrm{se},w}^{(i)}\right)\left\{\dot{g}_{\mathrm{sp},z}^{(i)}-\dot{g}_{\mathrm{sp},w}^{(i)}\right\} q_1^{(i)}q_0^{(i)};\end{aligned} \tag{S1}$$

where $\dot{g}_{\mathrm{se},x}^{(i)}$, $\dot{g}_{\mathrm{se},y}^{(i)}$, $\dot{g}_{\mathrm{se},z}^{(i)}$, and $\dot{g}_{\mathrm{se},w}^{(i)}$ are defined by the partial derivative of $g_{\mathrm{se}}^{(i)}(x,y,z,w)$ with respect to x, y, z, and w, respectively; $\dot{g}_{\mathrm{sp},x}^{(i)}$, $\dot{g}_{\mathrm{sp},y}^{(i)}$, $\dot{g}_{\mathrm{sp},z}^{(i)}$, and $\dot{g}_{\mathrm{sp},w}^{(i)}$ are defined in the same way for specificity; $\sigma_1^{(i)}(t)$ and $\sigma_0^{(i)}(t)$ are the limiting variances of $\hat{S}_1^{(i)}(t)$ and $\hat{S}_0^{(i)}(t)$, respectively. According to Section 3.2 in Hattori and Zhou, $\sigma_1^{(i)}(t)$ and $\sigma_0^{(i)}(t)$ in equation (S1) are consistently estimated by the Greenwood formula, in which the censoring distribution is estimated by using the median follow-up time. By replacing the theoretical quantities in equation (S1) with their consistent estimators, $\sigma_{\mathrm{se}}^{(i)}$, $\sigma_{\mathrm{sp}}^{(i)}$, and $\sigma_{\mathrm{se,sp}}^{(i)}$ can be consistently estimated, denoted by $\hat{\sigma}_{\mathrm{se}}^{(i)}$, $\hat{\sigma}_{\mathrm{sp}}^{(i)}$, and $\hat{\sigma}_{\mathrm{se,sp}}^{(i)}$, respectively, and the consistent estimator of $\mathbf{H}^{(i)}$ can be consequently obtained, denoted by $\hat{\mathbf{H}}^{(i)}$.

S2.2 | $\left\{\sigma^{(i)}_{\text{lnHR}}\right\}^2$: the asymptotic variance of $\hat{\mu}^{(i)}_{\text{lnHR}}$

In study i, we define $\tilde{Z}_j = \mathbf{1}\left(\tilde{X}_j > v^{(i)}\right)$ for simplicity, where $\mathbf{1}(\cdot)$ indicates the indicator function. Thus, $\tilde{Z}_j = 1$ if subject j belongs to the high expression group and $\tilde{Z}_j = 0$ if the low expression group, and $\tilde{Z}_j$ $\left(j = 1, \ldots, n^{(i)}\right)$ are the iid copies of $\tilde{Z}$. Let $\hat{\mu}^{(i)}_{\text{lnHR}}$ denote the maximum partial likelihood estimate from the Cox model

$$\lambda^{(i)}(t) = \lambda^{(i)}_0(t) \exp\left\{\mu^{(i)}_{\text{lnHR}} \tilde{Z}\right\}, \tag{S2}$$

where $\lambda^{(i)}(t)$ denotes the hazard function, $\lambda^{(i)}_0(t)$ the baseline hazard function.

Recall that $\tilde{Y}_j = \min\left(\tilde{T}_j, \tilde{C}_j\right)$ is the follow-up time; define $\tilde{\Delta}_j = \mathbf{1}\left(\tilde{T}_j \le \tilde{C}_j\right)$, where $\left(\tilde{Y}_j, \Delta_j\right)$ are iid copies of $\left(\tilde{Y}, \tilde{\Delta}\right)$; Define $N_j(u) = \mathbf{1}\left(\tilde{Y}_j \le u, \tilde{\Delta}_j = 1\right)$ the counting process, with $N_{0,j}(u) = \mathbf{1}\left(\tilde{Y}_j \le u, \tilde{\Delta}_j = 1, \tilde{X}_j \le v^{(i)}\right)$ and $N_{1,j}(u) = \mathbf{1}\left(\tilde{Y}_j \le u, \tilde{\Delta}_j = 1, \tilde{X}_j > v^{(i)}\right)$; define $Y_j(u) = \mathbf{1}\left(\tilde{Y}_j \ge u\right)$ the at-risk process, with $Y_{0,j}(u) = \mathbf{1}\left(\tilde{Y}_j \ge u, \tilde{X}_j \le v^{(i)}\right)$ and $Y_{1,j}(u) = \mathbf{1}\left(\tilde{Y}_j \ge u, \tilde{X}_j > v^{(i)}\right)$, and $0 \le u \le t$. The partial likelihood score function of the Cox model (S2) is

$$\mathbf{U}\left(\mu^{(i)}_{\text{lnHR}}\right) = \sum_{j=1}^{n^{(i)}} \int_0^t \tilde{Z}_j - \frac{E\left\{\tilde{Z} Y(u) e^{\mu^{(i)}_{\text{lnHR}} \tilde{Z}}\right\}}{E\left\{Y(u) e^{\mu^{(i)}_{\text{lnHR}} \tilde{Z}}\right\}} dM^{(i)}_{\text{cox},j}(u) \tag{S3}$$

with

$$\begin{aligned} dM^{(i)}_{\text{cox},j}(u) &= dN_j(u) - Y_j(u)\lambda^{(i)}_j(u) = \tilde{Z}_j dM^{(i)}_{1,j}(u) + \left(1 - \tilde{Z}_j\right) dM^{(i)}_{0,j}(u), \\ dM^{(i)}_{l,j}(u) &= dN_{l,j}(u) + Y_{l,j}(u) d \log S^{(i)}_l(u) \end{aligned} \tag{S4}$$

where $l = 0$ or 1 indicates the low or high expression group; $M^{(i)}_{\text{cox},j}(u)$, $M^{(i)}_{0,j}(u)$, and $M^{(i)}_{1,j}(u)$ indicates the martingales of the Cox model, the low, and the high expression group, respectively.

Let $\mathbf{I}\left(\mu^{(i)}_{\text{lnHR}}\right)$ denote the information matrix, and it holds that

$$\left\{n^{(i)}\right\}^{-1/2} \mathbf{U}\left(\mu^{(i)}_{\text{lnHR}}\right) \xrightarrow{D} N\left(0, \mathbf{I}\left(\mu^{(i)}_{\text{lnHR}}\right)\right). \tag{S5}$$

Denote the sample information at $\mu^{(i)}_{\text{lnHR}}$ to be $\hat{\mathbf{I}}\left(\mu^{(i)}_{\text{lnHR}}\right)$, and it can be shown that $\hat{\mathbf{I}}\left(\mu^{(i)}_{\text{lnHR}}\right) \big/ n^{(i)} \xrightarrow{P} \mathbf{I}\left(\mu^{(i)}_{\text{lnHR}}\right)$. To find the asymptotic normality of $\hat{\mu}^{(i)}_{\text{lnHR}}$, we expand $\mathbf{U}\left(\hat{\mu}^{(i)}_{\text{lnHR}}\right)$ about the true value $\mu^{(i)}_{\text{lnHR}}$:

$$\mathbf{U}\left(\hat{\mu}^{(i)}_{\text{lnHR}}\right) \simeq \mathbf{U}\left(\mu^{(i)}_{\text{lnHR}}\right) - \hat{\mathbf{I}}\left(\mu^{(i)}_{\text{lnHR}*}\right)\left\{\hat{\mu}^{(i)}_{\text{lnHR}} - \mu^{(i)}_{\text{lnHR}}\right\}$$

where $\left|\mu^{(i)}_{\text{lnHR}} - \mu^{(i)}_{\text{lnHR}*}\right| \le \left|\mu^{(i)}_{\text{lnHR}} - \hat{\mu}^{(i)}_{\text{lnHR}}\right|$. Since $\mathbf{U}\left(\hat{\mu}^{(i)}_{\text{lnHR}}\right) = 0$, we can obtain

$$\sqrt{n^{(i)}}\left\{\hat{\mu}^{(i)}_{\text{lnHR}} - \mu^{(i)}_{\text{lnHR}}\right\} \simeq \left\{\hat{\mathbf{I}}\left(\mu^{(i)}_{\text{lnHR}*}\right) \big/ n^{(i)}\right\}^{-1} \left\{n^{(i)}\right\}^{-1/2} \mathbf{U}\left(\mu^{(i)}_{\text{lnHR}}\right). \tag{S6}$$

Since $\hat{\mu}^{(i)}_{\text{lnHR}} \xrightarrow{P} \mu^{(i)}_{\text{lnHR}}$, which implies $\mu^{(i)}_{\text{lnHR}*} \xrightarrow{P} \mu^{(i)}_{\text{lnHR}}$, and $\hat{\mathbf{I}}\left(\mu^{(i)}_{\text{lnHR}*}\right) \big/ n^{(i)} \xrightarrow{P} \mathbf{I}\left(\mu^{(i)}_{\text{lnHR}}\right)$; with (S5) and Slutsky' theorem, it holds that

$$\sqrt{n^{(i)}}\left\{\hat{\mu}^{(i)}_{\text{lnHR}} - \mu^{(i)}_{\text{lnHR}}\right\} \xrightarrow{D} N\left(0, \left\{\sigma^{(i)}_{\text{lnHR}}\right\}^2\right), \text{ with } \left\{\sigma^{(i)}_{\text{lnHR}}\right\}^2 = \left\{\mathbf{I}\left(\mu^{(i)}_{\text{lnHR}}\right)\right\}^{-1}. \tag{S7}$$

Since $\hat{\mathbf{I}}\left(\hat{\mu}^{(i)}_{\text{lnHR}}\right) \big/ n^{(i)}$ is also a consistent estimator of $\mathbf{I}\left(\mu^{(i)}_{\text{lnHR}}\right)$, the asymptotic distribution in (S7) is unchanged if $\left\{\sigma^{(i)}_{\text{lnHR}}\right\}^2$ is replaced by $\left\{\hat{\sigma}^{(i)}_{\text{lnHR}}\right\}^2 = n^{(i)}\left\{\hat{\mathbf{I}}\left(\hat{\mu}^{(i)}_{\text{lnHR}}\right)\right\}^{-1}$. In practice, $\left\{\hat{\mathbf{I}}\left(\hat{\mu}^{(i)}_{\text{lnHR}}\right)\right\}^{-1/2}$ can be observed as the SE of lnHR in the prognosis studies.

S2.3 | $\sigma^{(i)}_{\text{se,lnHR}}$ and $\sigma^{(i)}_{\text{sp,lnHR}}$: the asymptotic covariances between $\left(\hat{\mu}^{(i)}_{\text{se}}, \hat{\mu}^{(i)}_{\text{lnHR}}\right)$ and $\left(\hat{\mu}^{(i)}_{\text{sp}}, \hat{\mu}^{(i)}_{\text{lnHR}}\right)$

In Appendix A of Hattori and Zhou, we knew that

$$\sqrt{n^{(i)}}\left\{\hat{\mu}^{(i)}_{\text{se}} - \mu^{(i)}_{\text{se}}\right\} \simeq \dot{g}^{(i)}_{\text{se},x}\sqrt{n^{(i)}/n^{(i)}_1} R^{(i)}_1 + \dot{g}^{(i)}_{\text{se},y}\sqrt{n^{(i)}/n^{(i)}_0} R^{(i)}_0 + \dot{g}^{(i)}_{\text{se},z} Q^{(i)}_1 + \dot{g}^{(i)}_{\text{se},w} Q^{(i)}_0,$$

where $R^{(i)}_l = \sqrt{n^{(i)}_l}\left\{\hat{S}^{(i)}_l(t) - S^{(i)}_l(t)\right\}$ and $Q^{(i)}_l = \sqrt{n^{(i)}}\left\{\hat{q}^{(i)}_l - q^{(i)}_l\right\}$ with $l = 0, 1$.

For simplicity, we denote $W^{(i)} = \sqrt{n^{(i)}}\left\{\hat{\mu}^{(i)}_{\text{lnHR}} - \mu^{(i)}_{\text{lnHR}}\right\}$, and the integrand in (S3) is denoted by $\tilde{Z}_j - \mathcal{E}$. Thus, the covariance between $\left(\hat{\mu}^{(i)}_{\text{se}}, \hat{\mu}^{(i)}_{\text{lnHR}}\right)$ can be written

$$
\begin{aligned}
&Cov\left(\sqrt{n^{(i)}}\left\{\hat{\mu}^{(i)}_{\text{se}} - \mu^{(i)}_{\text{se}}\right\}, \sqrt{n^{(i)}}\left\{\hat{\mu}^{(i)}_{\text{lnHR}} - \mu^{(i)}_{\text{lnHR}}\right\}\right) = Cov\left(\sqrt{n^{(i)}}\{\hat{\mu}^{(i)}_{\text{se}} - \mu^{(i)}_{\text{se}}\}, W^{(i)}\right)\\
\simeq&\dot{g}^{(i)}_{\text{se},x}\sqrt{n^{(i)}/n^{(i)}_1}Cov\left(R^{(i)}_1, W^{(i)}\right) + \dot{g}^{(i)}_{\text{se},y}\sqrt{n^{(i)}/n^{(i)}_0}Cov\left(R^{(i)}_0, W^{(i)}\right)\\
&+ \dot{g}^{(i)}_{\text{se},z}Cov\left(Q^{(i)}_1, W^{(i)}\right) + \dot{g}^{(i)}_{\text{se},w}Cov\left(Q^{(i)}_0, W^{(i)}\right).
\end{aligned}
\tag{S8}
$$

Given $S^{(i)}_1(t) > 0$, Hattori and Zhou defined

$$
R^{(i)}_1 = \sqrt{n^{(i)}_1}\left\{\hat{S}^{(i)}_1(t) - S^{(i)}_1(t)\right\} \simeq \frac{1}{\sqrt{n^{(i)}_1}}\sum_{i=1}^{n^{(i)}_1}\left\{-S^{(i)}_1(t)\int_0^t \frac{\hat{S}^{(i)}_1(u-)}{S^{(i)}_1(u)}\frac{dM^{(i)}_{1,j}(u)}{E\left\{Y_1(u)\right\}}\right\}.
$$

According to (S5)-(S7) and define $B^{(i)} = \hat{\mathbf{I}}\left(\hat{\mu}^{(i)}_{\text{lnHR}}\right)\Big/ n^{(i)}$, we can write

$$
W^{(i)} = \sqrt{n^{(i)}}\left\{\hat{\mu}^{(i)}_{\text{lnHR}} - \mu^{(i)}_{\text{lnHR}}\right\} \simeq \frac{1}{B^{(i)}}\frac{1}{\sqrt{n^{(i)}}}\sum_{j=1}^{n^{(i)}}\int_0^t \tilde{Z}_j - \mathcal{E}dM^{(i)}_{\text{cox},j}(u),
$$

where, both $R^{(i)}_1$ and $W^{(i)}$ are local square integrable martingales. Thus, we can write

$$
\begin{aligned}
Cov\left(R^{(i)}_1, W^{(i)}\right) &= E\left(R^{(i)}_1 W^{(i)}\right)\\
&\simeq \frac{-S^{(i)}_1(t)}{B^{(i)}}\frac{1}{\sqrt{n^{(i)}_1 n^{(i)}}}\sum_{j=1}^{n^{(i)}}E\left\{\int_0^t \frac{\tilde{Z} - \mathcal{E}}{E\left\{Y^{(i)}_1(u)\right\}}d\left\langle M^{(i)}_1, M^{(i)}_{cox}\right\rangle(u)\right\}.
\end{aligned}
$$

From (S4), we can obtain

$$
\begin{aligned}
d\left\langle M^{(i)}_{1,j}, M^{(i)}_{cox,j}\right\rangle(u) &\approx d\left\langle M^{(i)}_{1,j}, \tilde{Z}_j dM^{(i)}_{1,j} + \left(1 - \tilde{Z}_j\right)M^{(i)}_{0,j}\right\rangle(u)\\
&= \tilde{Z}_j d\left\langle M^{(i)}_{1,j}, M^{(i)}_{1,j}\right\rangle(u)\\
&= \tilde{Z}_j Y_j(u)dE\Lambda^{(i)}_1(u)\\
&= Y_{1,j}(u)d\Lambda^{(i)}_1(u). \qquad \left(\because \tilde{Z}_j Y_j(u) = Y_{1,j}(u)\right)
\end{aligned}
$$

Therefore,

$$
\begin{aligned}
Cov\left(R^{(i)}_1, W^{(i)}\right) &\simeq \frac{-S^{(i)}_1(t)}{B^{(i)}}\frac{1}{\sqrt{n^{(i)}_1 n^{(i)}}}\sum_{j=1}^{n^{(i)}}\int_0^t E\left\{\frac{\tilde{Z} - \mathcal{E}}{E\left\{Y_1(u)\right\}}Y_1(u)\right\}d\Lambda^{(i)}_1(u)\\
&\simeq \frac{-S^{(i)}_1(t)}{B^{(i)}}\sqrt{\frac{n^{(i)}}{n^{(i)}_1}}\int_0^t E\left\{\frac{E\left\{\tilde{Z}Y_1(u) - \mathcal{E}Y_1(u)\right\}}{E\left\{Y^{(i)}_1(u)\right\}}\right\}d\Lambda^{(i)}_1(u)\\
&= \frac{-S^{(i)}_1(t)}{B^{(i)}}\sqrt{\frac{n^{(i)}}{n^{(i)}_1}}\int_0^t 1 - \mathcal{E}d\Lambda^{(i)}_1(u) \qquad \left(\because \tilde{Z}_j Y_{1,j}(u) = Y_{1,j}(u)\right)\\
&\simeq \frac{S^{(i)}_1(t)}{B^{(i)}}\sqrt{\frac{n^{(i)}}{n^{(i)}_1}}\left\{\log S^{(i)}_1(t) - \int_0^t \frac{1}{S^{(i)}(u)}dS^{(i)}_1(u)\right\} \qquad \left(\because \mathcal{E} = \frac{S^{(i)}_1(u)}{S^{(i)}(u)}\right).
\end{aligned}
$$

Similarly, we can obtain

$$Cov\left(R_0^{(i)}, W^{(i)}\right) \simeq \frac{S_0^{(i)}(t)}{B^{(i)}}\sqrt{\frac{n^{(i)}}{n_0^{(i)}}}\left\{\log S_0^{(i)}(t) - \int_0^t \frac{1}{S^{(i)}(u)} dS_0^{(i)}(u)\right\}$$

Recall that $Q_1^{(i)} = \sqrt{n^{(i)}}\left\{\hat{q}_1^{(i)} - q_1^{(i)}\right\} \simeq \sqrt{n^{(i)}}E\left\{\tilde{Z} - q_1^{(i)}\right\}$. To find the covariance between $Q_1^{(i)}$ and $W^{(i)}$, we can write

$$\begin{aligned}
Cov\left(Q_1^{(i)}, W^{(i)}\right) &= E\left(Q_1^{(i)} W^{(i)}\right) \\
&\simeq \frac{1}{n^{(i)}}\frac{1}{B^{(i)}}\sum_{j=1}^{n^{(i)}} E\left\{E\left\{\tilde{Z} - q_1^{(i)}\right\}\int_0^t \tilde{Z} - \mathcal{E} dM_{cox,j}^{(i)}(u)\right\} \\
&\simeq \frac{1}{B^{(i)}}E\left[E\left\{\tilde{Z} - q_1^{(i)}\right\}E\left\{\int_0^t \tilde{Z} - \mathcal{E} dM_{cox,j}^{(i)}(u)\right\}\right] \\
&= 0
\end{aligned}$$

Similarly, $Cov\left(Q_0^{(i)}, W^{(i)}\right) \to 0$ as $n^{(i)} \to \infty$.

Finally, we let $\sigma_{\mathrm{se,lnHR}}^{(i)}$ denote the asymptotic covariances (S8) and it can be written by

$$\begin{aligned}
\sigma_{\mathrm{se,lnHR}}^{(i)} = \dot{g}_{\mathrm{se},x}^{(i)} &\frac{n^{(i)} S_1^{(i)}(t)}{n_1^{(i)} B^{(i)}}\left\{\log S_1^{(i)}(t) - \int_0^t \frac{1}{S^{(i)}(u)} dS_1^{(i)}(u)\right\} \\
+ \dot{g}_{\mathrm{se},y}^{(i)} &\frac{n^{(i)} S_0^{(i)}(t)}{n_0^{(i)} B^{(i)}}\left\{\log S_0^{(i)}(t) - \int_0^t \frac{1}{S^{(i)}(u)} dS_0^{(i)}(u)\right\}.
\end{aligned} \tag{S9}$$

In the similar way, let $\sigma_{\mathrm{sp,lnHR}}^{(i)}$ denote the asymptotic covariances between $\left(\hat{\mu}_{\mathrm{sp}}^{(i)}, \hat{\mu}_{\mathrm{lnHR}}^{(i)}\right)^{\top}$:

$$\begin{aligned}
\sigma_{\mathrm{sp,lnHR}}^{(i)} = \dot{g}_{\mathrm{sp},x}^{(i)} &\frac{n^{(i)} S_1^{(i)}(t)}{n_1^{(i)} B^{(i)}}\left\{\log S_1^{(i)}(t) - \int_0^t \frac{1}{S^{(i)}(u)} dS_1^{(i)}(u)\right\} \\
+ \dot{g}_{\mathrm{sp},y}^{(i)} &\frac{n^{(i)} S_0^{(i)}(t)}{n_0^{(i)} B^{(i)}}\left\{\log S_0^{(i)}(t) - \int_0^t \frac{1}{S^{(i)}(u)} dS_0^{(i)}(u)\right\}.
\end{aligned} \tag{S10}$$

By replacing $S_l^{(i)}$ with the observed KM estimators $\hat{S}_l^{(i)}$ $(l = 0, 1)$, we can obtain the consistent estimators of $\sigma_{\mathrm{se,lnHR}}^{(i)}$ and $\sigma_{\mathrm{sp,lnHR}}^{(i)}$, denoted by $\hat{\sigma}_{\mathrm{se,lnHR}}^{(i)}$ and $\hat{\sigma}_{\mathrm{sp,lnHR}}^{(i)}$, respectively. The integrations in the asymptotic covariances can be approximated by the trapezoidal rule:

$$\int_0^t \frac{1}{S^{(i)}(u)} dS_l^{(i)}(u) \approx \sum_{k=1}^{K}\left[\frac{1}{\hat{S}^{(i)}\left(t_k\right)} + \frac{1}{\hat{S}^{(i)}\left(t_{k-1}\right)}\right]\frac{\hat{S}_l^{(i)}\left(t_k\right) - \hat{S}_l^{(i)}\left(t_{k-1}\right)}{2}$$

where

$$\hat{S}^{(i)}(t) = \frac{n_0^{(i)}\hat{S}_0^{(i)}(t) + n_1^{(i)}\hat{S}_1^{(i)}(t)}{n^{(i)}}.$$

S3 | THE CONFIDENCE INTERVAL OF THE TIME-DEPENDENT SAUC

The variance of the time-dependent SAUC, denoted SAUC(t), can be constructed by the delta method. Define $\boldsymbol{D} = \int_0^1 \text{SROC}(t)\,\{1 - \text{SROC}(t)\}\,\nabla\text{SROC}(t)$, where ∇SROC(t) is the gradient of the linear function in SROC(t) with

$$\nabla\text{SROC}(t) = \left(1, \frac{-\rho_1\tau_{\text{se}}}{\tau_{\text{sp}}}, \frac{-\rho_1}{\tau_{\text{sp}}}\left\{\text{logit}(x) + \mu_{\text{se}}\right\}, \frac{-\rho_1\tau_{\text{se}}}{\tau_{\text{sp}}^2}\left\{\text{logit}(x) + \mu_{\text{se}}\right\}, \frac{-\tau_{\text{se}}}{\tau_{\text{sp}}}\left\{\text{logit}(x) + \mu_{\text{se}}\right\}\right)^{\top}.$$

Let $\hat{\boldsymbol{D}}$ denote the MLE of $\boldsymbol{D}$ by replacing the unknown parameters with their MLEs. By the delta method, the variance of the estimated SAUC(t) is consistently estimated by

$$Var\left[\text{SA}\hat{\text{U}}\text{C}(t)\right] = \hat{\boldsymbol{D}}^{\top}\hat{\boldsymbol{\Upsilon}}\hat{\boldsymbol{D}}.$$

For the HZ model (equation 4 in the main text), $\hat{\boldsymbol{\Upsilon}}$ is the estimated variance-covariance matrix of $\hat{\boldsymbol{\mu}}$ and $\hat{\boldsymbol{\Omega}}$. For the proposed method, $\hat{\boldsymbol{\Upsilon}}$ is the estimated variance-covariance matrix of $\hat{\boldsymbol{\theta}}$ and $\hat{\boldsymbol{\Psi}}$. By applying the delta method to the logit-transformed SAUC(t), the two-tailed confidence interval of the SA$\hat{\text{U}}$C(t) at significance level η is estimated by

$$\text{logit}^{-1}\left\{\text{logit}\left(\text{SA}\hat{\text{U}}\text{C}(t)\right) \pm z_{1-\eta/2}\frac{\sqrt{Var\left[\text{SA}\hat{\text{U}}\text{C}(t)\right]}}{\text{SA}\hat{\text{U}}\text{C}(t)\left[1 - \text{SA}\hat{\text{U}}\text{C}(t)\right]}\right\}.$$

S4 | THE ESTIMATIONS OF THE SROC(t) AND THE SAUC(t) IN KI67 EXAMPLE

As mentioned in Section 5, we present the funnel plot of studies reporting the KM curves in meta-analysis of Ki67.

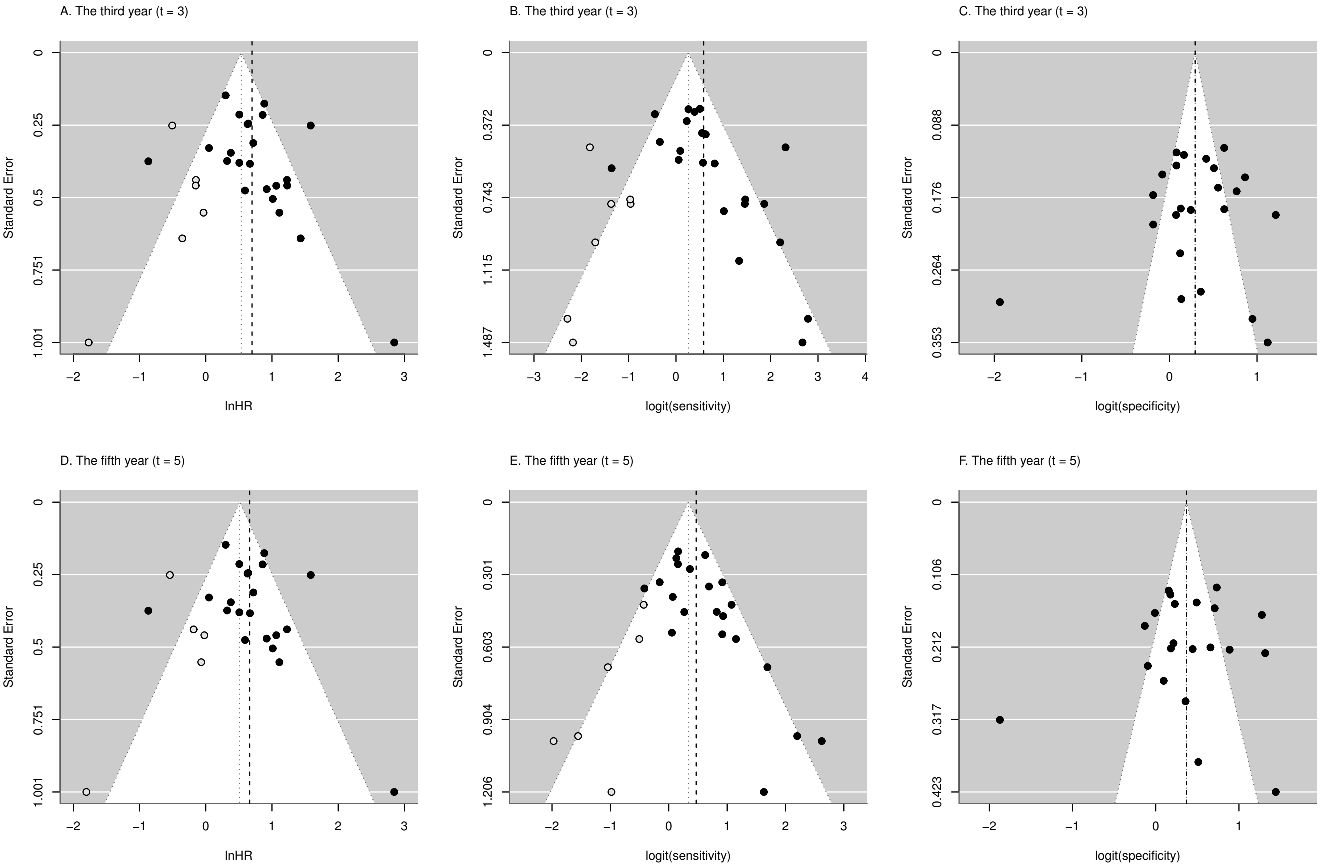

FIGURE S1 The funnel plots for studies reporting the KM curves in meta-analysis of Ki67. The circle points indicate the filled studies. The vertical black dashed lines are the lnHRs without reporting bias adjustment. The central axes of the funnel plots are adjusted lnHRs.

We present the estimates of other parameters at $t = 3$ and $t = 5$ by the HZ model ($p = 1$) and the proposed method ($p = 0.6, 0.4, 0.2$) in Table S4-S5, respectively.

The estimates of the SAUC(t) at $t = 3$ or $t = 5$ by the the HZ model ($p = 1$) and the proposed method ($p = 0.9, \dots, 0.1$) are presented in Table S6.

TABLE S4 Estimates of the other parameters for the SROC(3)

	$p = 1^*$	$p = 0.6$	$p = 0.4$	$p = 0.2$
μ_{se} (se)	0.670 (0.662)	0.681 (0.664)	0.702 (0.669)	0.764 (0.682)
μ_{sp} (sp)	0.282 (0.570)	0.233 (0.558)	0.168 (0.542)	0.064 (0.516)
$\mu_{\ln HR}$ (HR)		0.538 (1.713)	0.351 (1.420)	0.083 (1.087)
ψ_{se}	0.705	0.726	0.722	0.719
ψ_{sp}	0.51	0.511	0.518	0.528
$\psi_{\ln HR}$		0.467	0.519	0.571
ρ_1	-0.855	-0.854	-0.848	-0.846
ρ_2		0.416	0.469	0.527
ρ_3		-0.034	-0.092	-0.173
β		1.857	1.615	1.536
α		-0.944	-1.47	-1.888

ρ_1 denotes the correlation coefficient between μ_{se} and μ_{sp}; ρ_2 denotes that between μ_{se} and $\mu_{\ln HR}$; ρ_3 denotes that between μ_{sp} and $\mu_{\ln HR}$; $^*p = 1$ indicates estimates of the HZ model.

TABLE S5 Estimates of the other parameters for the SROC(5)

	$p = 1^*$	$p = 0.6$	$p = 0.4$	$p = 0.2$
μ_{se} (se)	0.526 (0.629)	0.513 (0.626)	0.512 (0.625)	0.538 (0.631)
μ_{sp} (sp)	0.354 (0.588)	0.295 (0.573)	0.224 (0.556)	0.107 (0.527)
$\mu_{\ln HR}$ (HR)		0.504 (1.655)	0.315 (1.370)	0.045 (1.046)
ψ_{se}	0.444	0.463	0.46	0.457
ψ_{sp}	0.58	0.582	0.589	0.6
$\psi_{\ln HR}$		0.451	0.511	0.568
ρ_1	-0.938	-0.9	-0.895	-0.897
ρ_2		0.377	0.436	0.503
ρ_3		0.147	0.064	-0.044
β		1.912	1.644	1.546
α		-0.91	-1.403	-1.795

ρ_1 denotes the correlation coefficient between μ_{se} and μ_{sp}; ρ_2 denotes that between μ_{se} and $\mu_{\ln HR}$; ρ_3 denotes that between μ_{sp} and $\mu_{\ln HR}$; $^*p = 1$ indicates estimates of the HZ model.

TABLE S6 The estimated SAUC(t) with 95% confidence intervals at $t = 3, 5$

	SAUC(3) (95% CI)	SAUC(5) (95% CI)
$p = 1^*$	0.649 (0.606, 0.690)	0.646 (0.610, 0.680)
$p = 0.9$	0.647 (0.605, 0.687)	0.644 (0.610, 0.678)
$p = 0.8$	0.644 (0.601, 0.685)	0.640 (0.604, 0.675)
$p = 0.7$	0.641 (0.597, 0.683)	0.636 (0.599, 0.672)
$p = 0.6$	0.638 (0.591, 0.682)	0.632 (0.592, 0.670)
$p = 0.5$	0.634 (0.583, 0.682)	0.627 (0.583, 0.669)
$p = 0.4$	0.631 (0.574, 0.684)	0.623 (0.573, 0.670)
$p = 0.3$	0.627 (0.562, 0.688)	0.618 (0.560, 0.672)
$p = 0.2$	0.624 (0.546, 0.695)	0.613 (0.545, 0.677)
$p = 0.1$	0.621 (0.526, 0.708)	0.608 (0.525, 0.685)

$^*p = 1$ indicates estimates of the HZ model.

S5 | SIMULATION PROCESS AND OPTIMIZATION DETAILS IN SIMULATION STUDIES

As mentioned in Section 6, we give more explanations about the data-generating process and estimation details in the simulation studies.

S5.1 | Data-generating process

Step 1: *Generating individual patient data in one prognosis study.*

The individual patient data (IPD) could be analyzed in each prognosis study but could not be accessed in meta-analysis. In each study, the total number of subjects was distributed from uniform distribution. With the sampled number of subjects, we generated the latent failure time ($\tilde{T}$) and censoring time ($\tilde{C}$); then, the time-to-event outcomes accessible in each prognosis study were the follow-up time $\tilde{Y} = \min(\tilde{T}, \tilde{C})$ with censoring indicator $\tilde{\Delta} = \mathbf{1}\left(\tilde{T}_j \leq \tilde{C}_j\right)$. The biomarker ($\tilde{X}$) and a cutoff value ($v'$) were generated based on the distributions in Table 2 of the main text; subjects with $\tilde{X} \leq v$ were grouped in $\tilde{Z} = 1$ and $\tilde{Z} = 0$ otherwise. The KM estimates of $S_0(t)$ and $S_1(t)$ at different time points were estimated using the R function `survfit()` in R package `survival`. The log-transformed hazard ratio (lnHR) and the corresponding standard error (SE) were estimated by the Cox regression implemented by the R function `coxph()` in R package `survival` with $\tilde{Z}$ as the covariate. The median of follow-up time took the median of $\tilde{Y}$.

Step 2: *Generating the observable data for meta-analysis.*

With the number of subjects in the different groups and the KM estimates, we could estimate the final logit-transformed sensitivity and specificity at $t = 2$. We considered the estimates at $t = 2$ because the biomarkers were generated based on whether $\tilde{T} \leq 2$ or not. When estimating the variances of the logit-transformed sensitivity and specificity, we estimated the distribution of $\tilde{C}$ by using the exponential distribution, regardless of the true distribution of $\tilde{C}$. Each prognosis study i would generate one list of data containing $\left(\hat{\mu}_{\text{se}}^{(i)}, \hat{\mu}_{\text{sp}}^{(i)}, \hat{\mu}_{\text{lnHR}}^{(i)}, \left\{\sigma_{\text{se}}^{(i)}\right\}^2, \left\{\sigma_{\text{sp}}^{(i)}\right\}^2, \left\{\sigma_{\text{lnHR}}^{(i)}\right\}^2, \sigma_{\text{se,sp}}^{(i)}, \sigma_{\text{se,lnHR}}^{(i)}, \sigma_{\text{sp,lnHR}}^{(i)}\right)$. In each simulation, we generated S number of prognosis studies as population studies for meta-analysis.

Step 3: *Generating selectively published data.*

We selectively chose N number of studies from S population studies to analogous the selective publication processes. Whether a study is published or not is distributed from the Bernoulli distribution with probability equal to $a(t_{\text{HR}}^{(i)}) = \Phi(\alpha + 5 \times t_{\text{HR}}^{(i)})$; Bernoulli random number 1 indicates published (selected) and 0 otherwise. Since we considered the marginal selection probability $p = 0.7$, the parameter α was solved from $0.7 = \sum_{i=1}^{S} a(t_{\text{HR}}^{(i)})$. We repeated the whole data-generating process (from generating IPD until selective publication) 1000 times. The final value of α was taken from the average of 1000 estimates. The estimated values of α were shown in Table S7; the biomarkers correspond to the scenarios in Table 2 of the main text.

TABLE S7 The values of α in different simulation scenarios.

Consor	p	Patients	Biomarker1	Biomarker2	Biomarker3	Biomarker4	Biomarker5	Biomarker6
$Exp(0.2)$	0.7	50-150	-14.14	-14.20	-4.00	-8.00	-7.91	-8.99
		40-300	-18.01	-18.02	-5.13	-10.24	-10.12	-11.52
	0.5	50-150	-11.06	-10.84	-1.28	-5.29	-5.21	-6.13
		40-300	-13.89	-13.50	-2.35	-7.31	-7.20	-8.28
$U[1,4]$	0.7	50-150	-13.68	-13.39	-2.17	-7.08	-6.96	-8.00
		40-300	-16.79	-16.27	-3.52	-9.52	-9.38	-10.58
LN	0.7	50-150	-12.71	-12.47	-1.80	-6.32	-6.23	-7.24
		40-300	-15.71	-15.27	-3.02	-8.59	-8.45	-9.64

Censor indicates the true censoring distribution.

S5.2 | Details in optimizing the likelihood function

The optimization of loglikelihood function (18) was implemented by R function `nlminb()`. The parameters to be estimated were $(\mu_{\text{se}}, \mu_{\text{sp}}, \mu_{\text{lnHR}}, \psi_{\text{se}}, \psi_{\text{sp}}, \psi_{\text{lnHR}}, \rho_1, \rho_2, \rho_3, \beta)$, where $\psi_{\text{se,sp}} = \psi_{\text{se}}\psi_{\text{sp}}\rho_1$, $\psi_{\text{se,lnHR}} = \psi_{\text{se}}\psi_{\text{lnHR}}\rho_2$, and $\psi_{\text{sp,lnHR}} = \psi_{\text{sp}}\psi_{\text{lnHR}}\rho_3$.

We set the initial values of estimating $(\mu_{\text{se}}, \mu_{\text{sp}}, \psi_{\text{se}}, \psi_{\text{se}}, \rho_1)$ as the ML estimates of the HZ model based on the published studies (BNM_O). The initial values of $(\mu_{\text{lnHR}}, \psi_{\text{lnHR}}, \rho_2, \rho_3)$ were randomly sampled from the uniform distribution $U[0.1, 0.5]$ with one digit. The initial values of β was randomly sampled from $U[4.5, 6.5]$. The constrains for $(\mu_{\text{se}}, \mu_{\text{sp}}, \mu_{\text{lnHR}})$ were in $[-3, 3]$, for $(\psi_{\text{se}}, \psi_{\text{sp}}, \psi_{\text{lnHR}})$ in $[0.001, 2]$, for ρ_1 in $[-0.999, -0.001]$, for (ρ_2, ρ_2) in $[-0.999, 0.999]$, and for β in $[0.001, 7]$.

S6 | ADDITIONAL ESTIMATION IN SIMULATION STUDIES

S6.1 | Summary of the estimated sensitivity and specificity

As mentioned Section 6 of the main text, we summarized estimated logit-transformed sensitivity and specificity by the proposed method corresponding to Table 2-3. The estimates were summarized in Table S8-S11.

TABLE S8 Comparison of medians with the first and third quantiles of estimates of SAUC(2) by the HZ model and the proposed method.

Patients	$S(N)$	B	Par	BNM_P	BNM_O	Proposed	RB	Bias
50-150	35 (25)	1	μ_{se}	1.98 (1.81, 2.15)	2.40 (2.20, 2.58)	2.29 (2.09, 2.49)	0.42	0.31
			μ_{sp}	0.47 (0.42, 0.52)	0.45 (0.39, 0.50)	0.44 (0.38, 0.50)	-0.02	-0.03
		2	μ_{se}	1.87 (1.65, 2.09)	2.38 (2.16, 2.60)	2.30 (2.07, 2.56)	0.51	0.44
			μ_{sp}	0.63 (0.54, 0.71)	0.55 (0.47, 0.64)	0.54 (0.45, 0.63)	-0.07	-0.09
		3	μ_{se}	0.27 (0.22, 0.32)	0.38 (0.32, 0.44)	0.33 (0.27, 0.39)	0.11	0.06
			μ_{sp}	0.21 (0.17, 0.25)	0.26 (0.22, 0.31)	0.22 (0.17, 0.27)	0.05	0.01
		4	μ_{se}	0.69 (0.63, 0.74)	0.79 (0.73, 0.85)	0.75 (0.68, 0.81)	0.11	0.06
			μ_{sp}	0.31 (0.27, 0.35)	0.37 (0.33, 0.42)	0.33 (0.28, 0.39)	0.06	0.02
		5	μ_{se}	0.68 (0.61, 0.75)	0.80 (0.72, 0.87)	0.74 (0.66, 0.82)	0.12	0.06
			μ_{sp}	0.31 (0.27, 0.36)	0.36 (0.30, 0.42)	0.32 (0.27, 0.38)	0.05	0.01
		6	μ_{se}	0.53 (0.48, 0.58)	0.59 (0.54, 0.65)	0.55 (0.49, 0.61)	0.06	0.02
			μ_{sp}	0.56 (0.50, 0.61)	0.67 (0.61, 0.73)	0.61 (0.55, 0.68)	0.11	0.05
	70 (49)	1	μ_{se}	1.99 (1.87, 2.12)	2.42 (2.28, 2.55)	2.30 (2.16, 2.46)	0.42	0.31
			μ_{sp}	0.47 (0.43, 0.50)	0.45 (0.41, 0.48)	0.44 (0.41, 0.48)	-0.02	-0.03
		2	μ_{se}	1.89 (1.73, 2.04)	2.40 (2.25, 2.55)	2.30 (2.14, 2.48)	0.51	0.41
			μ_{sp}	0.61 (0.56, 0.67)	0.54 (0.49, 0.59)	0.53 (0.47, 0.59)	-0.07	-0.09
		3	μ_{se}	0.27 (0.24, 0.31)	0.38 (0.35, 0.43)	0.32 (0.28, 0.36)	0.11	0.05
			μ_{sp}	0.21 (0.18, 0.24)	0.26 (0.23, 0.30)	0.21 (0.17, 0.24)	0.05	-0.00
		4	μ_{se}	0.68 (0.65, 0.72)	0.79 (0.74, 0.83)	0.73 (0.69, 0.78)	0.10	0.05
			μ_{sp}	0.31 (0.29, 0.34)	0.37 (0.34, 0.40)	0.32 (0.29, 0.36)	0.06	0.01
		5	μ_{se}	0.69 (0.64, 0.73)	0.80 (0.75, 0.86)	0.73 (0.68, 0.79)	0.12	0.05
			μ_{sp}	0.31 (0.28, 0.35)	0.36 (0.32, 0.40)	0.31 (0.27, 0.36)	0.05	0.00
		6	μ_{se}	0.53 (0.49, 0.56)	0.59 (0.55, 0.63)	0.54 (0.50, 0.58)	0.06	0.01
			μ_{sp}	0.56 (0.52, 0.60)	0.67 (0.63, 0.71)	0.60 (0.55, 0.65)	0.11	0.04
	200 (140)	1	μ_{se}	2.00 (1.93, 2.07)	2.42 (2.35, 2.49)	2.29 (2.21, 2.38)	0.42	0.30
			μ_{sp}	0.47 (0.44, 0.49)	0.44 (0.42, 0.46)	0.44 (0.41, 0.46)	-0.03	-0.03
		2	μ_{se}	1.88 (1.79, 1.97)	2.36 (2.28, 2.47)	2.26 (2.17, 2.38)	0.48	0.39
			μ_{sp}	0.62 (0.59, 0.65)	0.55 (0.51, 0.58)	0.54 (0.50, 0.58)	-0.07	-0.08
		3	μ_{se}	0.27 (0.25, 0.29)	0.38 (0.36, 0.41)	0.32 (0.29, 0.35)	0.11	0.05
			μ_{sp}	0.21 (0.19, 0.23)	0.26 (0.24, 0.28)	0.20 (0.18, 0.23)	0.05	-0.01
		4	μ_{se}	0.69 (0.66, 0.71)	0.79 (0.76, 0.82)	0.73 (0.70, 0.76)	0.11	0.05
			μ_{sp}	0.31 (0.30, 0.33)	0.37 (0.35, 0.39)	0.32 (0.30, 0.34)	0.06	0.01
		5	μ_{se}	0.69 (0.66, 0.72)	0.80 (0.77, 0.84)	0.73 (0.69, 0.76)	0.12	0.04
			μ_{sp}	0.31 (0.29, 0.33)	0.36 (0.34, 0.38)	0.31 (0.29, 0.34)	0.05	0.00
		6	μ_{se}	0.53 (0.51, 0.55)	0.59 (0.57, 0.61)	0.53 (0.51, 0.56)	0.06	0.01
			μ_{sp}	0.55 (0.53, 0.58)	0.66 (0.64, 0.69)	0.60 (0.57, 0.63)	0.11	0.04

B denotes the scenarios of biomarker coresponding to Table 2; CR shows convergence rate of the proposed method; estimates are summarized by median (first quantile, third quantiles); RB denotes reporting bias; Bias denotes bias of the proposed method.

TABLE S9 Comparison of medians with the first and third quantiles of estimates of SAUC(2) by the HZ model and the proposed method.

Patients	$S(N)$	B	Par	BNM$_P$	BNM$_O$	Proposed	RB	Bias
40-300	35 (25)	1	μ_{se}	1.99 (1.82, 2.15)	2.36 (2.17, 2.54)	2.23 (2.02, 2.45)	0.37	0.25
			μ_{sp}	0.48 (0.44, 0.53)	0.43 (0.39, 0.48)	0.45 (0.40, 0.49)	-0.05	-0.04
		2	μ_{se}	1.93 (1.71, 2.15)	2.43 (2.21, 2.66)	2.33 (2.09, 2.63)	0.50	0.40
			μ_{sp}	0.64 (0.56, 0.72)	0.54 (0.46, 0.61)	0.55 (0.46, 0.63)	-0.11	-0.09
		3	μ_{se}	0.28 (0.24, 0.33)	0.37 (0.32, 0.42)	0.33 (0.28, 0.38)	0.08	0.04
			μ_{sp}	0.22 (0.18, 0.25)	0.25 (0.21, 0.29)	0.21 (0.17, 0.25)	0.03	-0.00
		4	μ_{se}	0.70 (0.65, 0.74)	0.77 (0.72, 0.82)	0.73 (0.68, 0.79)	0.07	0.03
			μ_{sp}	0.32 (0.29, 0.35)	0.35 (0.31, 0.39)	0.33 (0.29, 0.38)	0.03	0.01
		5	μ_{se}	0.70 (0.64, 0.76)	0.78 (0.71, 0.85)	0.74 (0.67, 0.81)	0.08	0.04
			μ_{sp}	0.32 (0.28, 0.37)	0.35 (0.29, 0.41)	0.33 (0.27, 0.38)	0.03	0.01
		6	μ_{se}	0.54 (0.50, 0.58)	0.56 (0.51, 0.61)	0.55 (0.50, 0.60)	0.02	0.01
			μ_{sp}	0.57 (0.52, 0.63)	0.67 (0.61, 0.73)	0.62 (0.55, 0.68)	0.10	0.04
	70 (49)	1	μ_{se}	2.01 (1.88, 2.13)	2.38 (2.24, 2.50)	2.26 (2.12, 2.40)	0.37	0.25
			μ_{sp}	0.48 (0.45, 0.51)	0.43 (0.39, 0.47)	0.45 (0.41, 0.48)	-0.05	-0.03
		2	μ_{se}	1.91 (1.76, 2.07)	2.41 (2.26, 2.57)	2.28 (2.11, 2.47)	0.50	0.37
			μ_{sp}	0.65 (0.59, 0.70)	0.54 (0.49, 0.60)	0.56 (0.50, 0.62)	-0.11	-0.09
		3	μ_{se}	0.28 (0.25, 0.31)	0.37 (0.33, 0.40)	0.32 (0.28, 0.35)	0.09	0.04
			μ_{sp}	0.22 (0.19, 0.24)	0.25 (0.22, 0.27)	0.21 (0.18, 0.24)	0.03	-0.00
		4	μ_{se}	0.70 (0.67, 0.73)	0.77 (0.73, 0.81)	0.73 (0.70, 0.77)	0.07	0.03
			μ_{sp}	0.32 (0.30, 0.34)	0.35 (0.32, 0.38)	0.33 (0.30, 0.36)	0.03	0.01
		5	μ_{se}	0.70 (0.66, 0.74)	0.78 (0.73, 0.83)	0.73 (0.68, 0.78)	0.08	0.03
			μ_{sp}	0.32 (0.29, 0.35)	0.35 (0.31, 0.38)	0.33 (0.29, 0.37)	0.03	0.01
		6	μ_{se}	0.54 (0.51, 0.57)	0.57 (0.53, 0.60)	0.55 (0.51, 0.59)	0.02	0.01
			μ_{sp}	0.57 (0.53, 0.61)	0.67 (0.62, 0.71)	0.61 (0.57, 0.66)	0.10	0.04
	200 (140)	1	μ_{se}	2.01 (1.93, 2.08)	2.38 (2.30, 2.46)	2.26 (2.17, 2.36)	0.38	0.25
			μ_{sp}	0.48 (0.46, 0.50)	0.43 (0.41, 0.45)	0.45 (0.43, 0.47)	-0.05	-0.03
		2	μ_{se}	1.91 (1.81, 2.01)	2.40 (2.31, 2.51)	2.27 (2.16, 2.39)	0.49	0.35
			μ_{sp}	0.65 (0.61, 0.68)	0.54 (0.51, 0.58)	0.57 (0.53, 0.60)	-0.10	-0.08
		3	μ_{se}	0.28 (0.26, 0.30)	0.36 (0.34, 0.39)	0.31 (0.29, 0.34)	0.09	0.03
			μ_{sp}	0.22 (0.20, 0.23)	0.25 (0.23, 0.26)	0.21 (0.19, 0.23)	0.03	-0.01
		4	μ_{se}	0.70 (0.68, 0.72)	0.77 (0.74, 0.79)	0.73 (0.70, 0.76)	0.07	0.03
			μ_{sp}	0.32 (0.31, 0.34)	0.35 (0.33, 0.37)	0.33 (0.31, 0.35)	0.03	0.01
		5	μ_{se}	0.70 (0.67, 0.72)	0.78 (0.75, 0.81)	0.73 (0.69, 0.76)	0.08	0.03
			μ_{sp}	0.32 (0.30, 0.34)	0.35 (0.33, 0.37)	0.33 (0.31, 0.35)	0.03	0.01
		6	μ_{se}	0.54 (0.52, 0.56)	0.56 (0.54, 0.58)	0.54 (0.52, 0.56)	0.02	0.00
			μ_{sp}	0.57 (0.55, 0.59)	0.67 (0.65, 0.70)	0.61 (0.59, 0.64)	0.10	0.04

B denotes the scenarios of biomarker coresponding to Table 2; CR shows convergence rate of the proposed method; estimates are summarized by median (first quantile, third quantiles); RB denotes reporting bias; Bias denotes bias of the proposed method.

TABLE S10 Comparison of medians with the first and third quantiles of estimates of SAUC(2) by the HZ model and the proposed method.

Patients	$S(N)$	B	Par	BNM_P	BNM_O	Proposed	RB	Bias
50-150	50 (25)	1	μ_{se}	1.98 (1.84, 2.13)	2.52 (2.33, 2.70)	2.31 (2.10, 2.53)	0.53	0.32
			μ_{sp}	0.47 (0.43, 0.51)	0.47 (0.42, 0.52)	0.44 (0.38, 0.50)	0.00	-0.02
		2	μ_{se}	1.87 (1.68, 2.06)	2.45 (2.24, 2.66)	2.27 (2.03, 2.57)	0.58	0.40
			μ_{sp}	0.62 (0.55, 0.70)	0.59 (0.51, 0.66)	0.56 (0.47, 0.65)	-0.03	-0.06
		3	μ_{se}	0.27 (0.23, 0.32)	0.45 (0.40, 0.51)	0.33 (0.27, 0.40)	0.18	0.06
			μ_{sp}	0.21 (0.18, 0.24)	0.29 (0.25, 0.34)	0.20 (0.14, 0.26)	0.08	-0.01
		4	μ_{se}	0.68 (0.64, 0.73)	0.85 (0.79, 0.91)	0.74 (0.67, 0.81)	0.17	0.05
			μ_{sp}	0.31 (0.28, 0.35)	0.41 (0.36, 0.45)	0.32 (0.27, 0.37)	0.09	0.01
		5	μ_{se}	0.68 (0.63, 0.74)	0.86 (0.78, 0.94)	0.73 (0.65, 0.81)	0.18	0.04
			μ_{sp}	0.31 (0.27, 0.35)	0.40 (0.35, 0.46)	0.31 (0.25, 0.37)	0.09	-0.00
		6	μ_{se}	0.53 (0.49, 0.57)	0.62 (0.56, 0.68)	0.53 (0.47, 0.59)	0.10	-0.00
			μ_{sp}	0.55 (0.51, 0.60)	0.74 (0.68, 0.80)	0.61 (0.55, 0.69)	0.19	0.06
	100 (50)	1	μ_{se}	1.99 (1.88, 2.10)	2.53 (2.42, 2.66)	2.30 (2.15, 2.45)	0.54	0.31
			μ_{sp}	0.47 (0.44, 0.50)	0.47 (0.43, 0.50)	0.44 (0.41, 0.48)	-0.00	-0.03
		2	μ_{se}	1.88 (1.75, 2.03)	2.46 (2.31, 2.61)	2.28 (2.12, 2.48)	0.57	0.40
			μ_{sp}	0.62 (0.57, 0.66)	0.58 (0.53, 0.63)	0.55 (0.49, 0.61)	-0.03	-0.06
		3	μ_{se}	0.27 (0.24, 0.30)	0.45 (0.41, 0.49)	0.32 (0.29, 0.37)	0.18	0.05
			μ_{sp}	0.21 (0.19, 0.23)	0.29 (0.26, 0.32)	0.19 (0.16, 0.23)	0.08	-0.02
		4	μ_{se}	0.68 (0.66, 0.72)	0.85 (0.80, 0.89)	0.73 (0.68, 0.77)	0.16	0.04
			μ_{sp}	0.31 (0.29, 0.34)	0.40 (0.37, 0.44)	0.32 (0.28, 0.35)	0.09	0.00
		5	μ_{se}	0.69 (0.65, 0.73)	0.86 (0.81, 0.91)	0.72 (0.67, 0.78)	0.18	0.04
			μ_{sp}	0.31 (0.28, 0.34)	0.40 (0.36, 0.43)	0.30 (0.26, 0.34)	0.08	-0.01
		6	μ_{se}	0.53 (0.50, 0.55)	0.62 (0.58, 0.66)	0.52 (0.48, 0.56)	0.09	-0.01
			μ_{sp}	0.56 (0.52, 0.59)	0.74 (0.70, 0.79)	0.61 (0.56, 0.66)	0.19	0.05
	300 (150)	1	μ_{se}	2.00 (1.94, 2.06)	2.53 (2.47, 2.61)	2.32 (2.23, 2.41)	0.54	0.32
			μ_{sp}	0.47 (0.45, 0.48)	0.46 (0.44, 0.48)	0.44 (0.42, 0.46)	-0.00	-0.03
		2	μ_{se}	1.87 (1.81, 1.94)	2.40 (2.32, 2.53)	2.23 (2.13, 2.38)	0.53	0.36
			μ_{sp}	0.62 (0.59, 0.65)	0.59 (0.55, 0.62)	0.56 (0.52, 0.60)	-0.03	-0.06
		3	μ_{se}	0.27 (0.25, 0.29)	0.45 (0.43, 0.47)	0.33 (0.30, 0.35)	0.18	0.05
			μ_{sp}	0.21 (0.20, 0.22)	0.29 (0.28, 0.31)	0.19 (0.17, 0.21)	0.08	-0.02
		4	μ_{se}	0.68 (0.67, 0.70)	0.85 (0.82, 0.88)	0.73 (0.70, 0.76)	0.17	0.04
			μ_{sp}	0.31 (0.30, 0.33)	0.40 (0.39, 0.42)	0.31 (0.29, 0.33)	0.09	-0.00
		5	μ_{se}	0.69 (0.66, 0.71)	0.87 (0.83, 0.90)	0.72 (0.69, 0.76)	0.18	0.03
			μ_{sp}	0.31 (0.29, 0.33)	0.40 (0.37, 0.42)	0.30 (0.28, 0.32)	0.09	-0.01
		6	μ_{se}	0.53 (0.51, 0.54)	0.62 (0.60, 0.64)	0.52 (0.49, 0.54)	0.09	-0.01
			μ_{sp}	0.55 (0.53, 0.57)	0.74 (0.72, 0.77)	0.61 (0.58, 0.63)	0.19	0.05

B denotes the scenarios of biomarker coresponding to Table 2; CR shows convergence rate of the proposed method; estimates are summarized by median (first quantile, third quantiles); RB denotes reporting bias; Bias denotes bias of the proposed method.

TABLE S11 Comparison of medians with the first and third quantiles of estimates of SAUC(2) by the HZ model and the proposed method.

Patients	$S(N)$	B	Par	BNM_P	BNM_O	Proposed	RB	Bias
40-300	50 (25)	1	μ_{se}	1.99 (1.84, 2.14)	2.47 (2.28, 2.65)	2.22 (1.99, 2.45)	0.48	0.23
			μ_{sp}	0.48 (0.45, 0.52)	0.43 (0.39, 0.48)	0.44 (0.39, 0.49)	-0.05	-0.04
		2	μ_{se}	1.93 (1.74, 2.11)	2.49 (2.28, 2.71)	2.27 (2.00, 2.57)	0.56	0.34
			μ_{sp}	0.64 (0.58, 0.70)	0.55 (0.48, 0.62)	0.57 (0.48, 0.64)	-0.09	-0.07
		3	μ_{se}	0.28 (0.24, 0.32)	0.42 (0.37, 0.47)	0.33 (0.28, 0.39)	0.14	0.05
			μ_{sp}	0.22 (0.19, 0.25)	0.27 (0.23, 0.31)	0.20 (0.16, 0.24)	0.05	-0.02
		4	μ_{se}	0.70 (0.66, 0.74)	0.81 (0.76, 0.86)	0.73 (0.67, 0.79)	0.11	0.03
			μ_{sp}	0.32 (0.29, 0.35)	0.37 (0.33, 0.41)	0.32 (0.28, 0.36)	0.06	0.00
		5	μ_{se}	0.70 (0.65, 0.75)	0.82 (0.75, 0.89)	0.74 (0.66, 0.82)	0.12	0.04
			μ_{sp}	0.32 (0.28, 0.36)	0.37 (0.32, 0.42)	0.32 (0.27, 0.37)	0.05	-0.00
		6	μ_{se}	0.54 (0.51, 0.57)	0.57 (0.53, 0.62)	0.53 (0.48, 0.58)	0.03	-0.01
			μ_{sp}	0.57 (0.53, 0.62)	0.74 (0.68, 0.79)	0.62 (0.56, 0.70)	0.17	0.05
	100 (50)	1	μ_{se}	2.01 (1.90, 2.11)	2.48 (2.36, 2.60)	2.22 (2.05, 2.39)	0.48	0.21
			μ_{sp}	0.48 (0.45, 0.51)	0.43 (0.40, 0.46)	0.45 (0.41, 0.48)	-0.05	-0.04
		2	μ_{se}	1.91 (1.79, 2.04)	2.48 (2.35, 2.63)	2.23 (2.05, 2.44)	0.57	0.32
			μ_{sp}	0.65 (0.60, 0.69)	0.55 (0.50, 0.60)	0.58 (0.52, 0.64)	-0.09	-0.07
		3	μ_{se}	0.28 (0.25, 0.31)	0.42 (0.39, 0.46)	0.32 (0.29, 0.37)	0.14	0.05
			μ_{sp}	0.22 (0.20, 0.24)	0.26 (0.24, 0.29)	0.20 (0.17, 0.23)	0.05	-0.02
		4	μ_{se}	0.70 (0.67, 0.73)	0.81 (0.77, 0.84)	0.73 (0.69, 0.77)	0.11	0.03
			μ_{sp}	0.32 (0.30, 0.34)	0.38 (0.35, 0.40)	0.32 (0.29, 0.36)	0.06	0.00
		5	μ_{se}	0.70 (0.67, 0.73)	0.82 (0.77, 0.87)	0.73 (0.67, 0.78)	0.12	0.03
			μ_{sp}	0.32 (0.29, 0.35)	0.37 (0.33, 0.40)	0.32 (0.28, 0.36)	0.05	0.00
		6	μ_{se}	0.54 (0.52, 0.57)	0.57 (0.54, 0.61)	0.53 (0.50, 0.57)	0.03	-0.01
			μ_{sp}	0.57 (0.54, 0.60)	0.74 (0.70, 0.78)	0.63 (0.58, 0.67)	0.17	0.05
	300 (151)	1	μ_{se}	2.01 (1.94, 2.07)	2.49 (2.41, 2.56)	2.24 (2.14, 2.34)	0.48	0.23
			μ_{sp}	0.48 (0.46, 0.50)	0.43 (0.41, 0.45)	0.45 (0.43, 0.47)	-0.05	-0.03
		2	μ_{se}	1.92 (1.84, 1.99)	2.45 (2.38, 2.57)	2.23 (2.12, 2.38)	0.54	0.31
			μ_{sp}	0.64 (0.62, 0.67)	0.56 (0.52, 0.59)	0.59 (0.54, 0.62)	-0.09	-0.06
		3	μ_{se}	0.28 (0.26, 0.29)	0.41 (0.39, 0.44)	0.32 (0.30, 0.35)	0.14	0.04
			μ_{sp}	0.22 (0.20, 0.23)	0.27 (0.25, 0.28)	0.20 (0.18, 0.21)	0.05	-0.02
		4	μ_{se}	0.70 (0.68, 0.71)	0.81 (0.79, 0.83)	0.73 (0.71, 0.75)	0.11	0.03
			μ_{sp}	0.32 (0.31, 0.33)	0.38 (0.36, 0.39)	0.32 (0.30, 0.34)	0.06	0.00
		5	μ_{se}	0.70 (0.68, 0.72)	0.82 (0.79, 0.84)	0.72 (0.69, 0.75)	0.12	0.03
			μ_{sp}	0.32 (0.31, 0.34)	0.37 (0.35, 0.39)	0.33 (0.30, 0.35)	0.05	0.00
		6	μ_{se}	0.54 (0.53, 0.55)	0.57 (0.55, 0.59)	0.53 (0.51, 0.55)	0.03	-0.01
			μ_{sp}	0.57 (0.56, 0.59)	0.74 (0.72, 0.76)	0.62 (0.59, 0.65)	0.16	0.05

B denotes the scenarios of biomarker coresponding to Table 2; CR shows convergence rate of the proposed method; estimates are summarized by median (first quantile, third quantiles); RB denotes reporting bias; Bias denotes bias of the proposed method.

S6.2 | The estimates of the proposed method when censoring distribution was misfitted

As mentioned at the end of Section 6 of the main text, we present the summarized estimates of the proposed method when the censoring distribution was misfitted by the exponential distribution. In the simulation studies, we considered the situation when $p = 0.7$. The estimates were summarized in Table S12-S13.

TABLE S12 Summary of estimates of SAUC(2) by the HZ model and the proposed method when the true censoring distribution follows uniform distribution $U[1,4]$ and is incorrectly estimated by the exponential distribution.

Patients	$S(N)$	B	BNM_P	BNM_O	Proposed	CR	RB	Bias
50-150	35 (25)	1	71.00 (69.82, 72.14)	72.73 (70.99, 74.25)	72.51 (70.49, 74.46)	64	1.74	1.51
		2	76.85 (75.85, 77.97)	78.42 (76.95, 79.73)	78.54 (76.86, 80.04)	56.9	1.57	1.68
		3	57.70 (56.82, 58.64)	60.37 (59.60, 61.19)	58.62 (57.72, 59.70)	61.2	2.67	0.93
		4	65.26 (64.11, 66.38)	67.77 (66.62, 68.86)	66.72 (65.34, 67.91)	61.1	2.50	1.45
		5	65.32 (64.41, 66.19)	67.85 (67.01, 68.65)	66.81 (65.64, 67.77)	55.5	2.53	1.48
		6	67.02 (66.15, 67.86)	69.71 (68.84, 70.52)	68.23 (67.03, 69.32)	57.7	2.69	1.21
	70 (49)	1	70.95 (70.13, 71.74)	72.57 (71.38, 73.67)	72.32 (70.89, 73.82)	81.6	1.61	1.37
		2	76.92 (76.13, 77.68)	78.45 (77.47, 79.53)	78.50 (77.32, 79.75)	76.6	1.53	1.58
		3	57.83 (57.21, 58.35)	60.46 (59.82, 61.05)	58.73 (57.99, 59.43)	73	2.64	0.90
		4	65.33 (64.45, 66.05)	67.79 (66.95, 68.54)	66.65 (65.69, 67.44)	72.4	2.46	1.31
		5	65.21 (64.59, 65.88)	67.75 (67.02, 68.37)	66.44 (65.62, 67.29)	66.8	2.54	1.23
		6	67.15 (66.53, 67.67)	69.80 (69.15, 70.39)	68.23 (67.44, 68.91)	68.3	2.65	1.07
	200 (140)	1	70.80 (70.31, 71.29)	72.45 (71.77, 73.05)	72.09 (71.21, 73.09)	90	1.64	1.29
		2	76.94 (76.44, 77.38)	78.28 (77.58, 79.00)	78.30 (77.54, 79.07)	86.4	1.34	1.36
		3	57.86 (57.52, 58.17)	60.48 (60.17, 60.82)	58.63 (58.19, 59.08)	83.2	2.62	0.77
		4	65.26 (64.75, 65.72)	67.73 (67.24, 68.22)	66.54 (66.01, 67.02)	83.9	2.46	1.28
		5	65.18 (64.75, 65.55)	67.67 (67.22, 68.08)	66.30 (65.78, 66.83)	64.9	2.49	1.11
		6	67.11 (66.76, 67.47)	69.79 (69.46, 70.12)	68.00 (67.56, 68.45)	75	2.68	0.89
40-300	35 (24)	1	71.40 (70.57, 72.35)	72.30 (71.01, 73.54)	71.83 (70.50, 73.30)	61.8	0.90	0.43
		2	77.10 (76.34, 77.90)	77.83 (76.80, 79.00)	77.57 (76.33, 78.78)	52.9	0.73	0.48
		3	58.05 (57.35, 58.66)	59.87 (59.27, 60.52)	58.77 (57.92, 59.59)	47.8	1.82	0.72
		4	65.57 (64.73, 66.36)	67.12 (66.26, 68.00)	66.66 (65.73, 67.70)	51	1.54	1.09
		5	65.60 (64.84, 66.31)	67.18 (66.44, 67.91)	66.58 (65.67, 67.44)	44	1.58	0.98
		6	67.39 (66.65, 68.09)	69.11 (68.37, 69.83)	68.12 (67.17, 68.99)	50.8	1.71	0.73
	70 (49)	1	71.26 (70.62, 71.93)	72.02 (71.09, 72.94)	71.77 (70.68, 72.72)	79.8	0.77	0.51
		2	77.25 (76.63, 77.78)	77.91 (77.15, 78.72)	77.66 (76.73, 78.50)	73.3	0.66	0.42
		3	58.03 (57.63, 58.49)	59.87 (59.42, 60.34)	58.69 (58.12, 59.20)	59.2	1.84	0.66
		4	65.63 (65.08, 66.22)	67.17 (66.58, 67.74)	66.60 (65.90, 67.22)	63.7	1.53	0.96
		5	65.54 (65.03, 66.03)	67.15 (66.64, 67.71)	66.43 (65.72, 67.10)	58.2	1.61	0.89
		6	67.41 (66.92, 67.86)	69.08 (68.61, 69.57)	68.05 (67.27, 68.64)	63.6	1.67	0.64
	200 (139)	1	71.26 (70.88, 71.62)	71.98 (71.38, 72.52)	71.70 (71.10, 72.40)	87.4	0.72	0.45
		2	77.19 (76.86, 77.56)	77.95 (77.49, 78.48)	77.66 (77.19, 78.29)	79.8	0.76	0.47
		3	57.99 (57.74, 58.27)	59.89 (59.63, 60.16)	58.64 (58.29, 59.00)	64.7	1.90	0.65
		4	65.59 (65.19, 65.91)	67.14 (66.79, 67.48)	66.57 (66.15, 66.96)	64.4	1.55	0.98
		5	65.57 (65.29, 65.88)	67.16 (66.83, 67.45)	66.32 (65.94, 66.69)	56	1.58	0.75
		6	67.42 (67.14, 67.72)	69.12 (68.86, 69.43)	67.95 (67.55, 68.42)	67	1.70	0.53

B denotes the scenarios of biomarker coresponding to Table 2; CR shows convergence rate of the proposed method; estimates are summarized by median (first quantile, third quantiles); RB denotes reporting bias; Bias denotes bias of the proposed method.

TABLE S13 Summary of estimates of SAUC(2) by the HZ model and the proposed method when the true censoring distribution follows lognormal distribution $LN(1.5\log 2, (\log 2)^2)$ and is incorrectly estimated by the exponential distribution.

Patients	$S(N)$	B	BNM_P	BNM_O	Proposed	CR	RB	Bias
50-150	35 (25)	1	71.02 (69.84, 72.12)	72.56 (70.77, 74.23)	72.90 (70.65, 74.86)	50.3	1.54	1.87
		2	76.75 (75.78, 77.83)	78.14 (76.80, 79.62)	78.17 (76.63, 80.11)	44.3	1.39	1.42
		3	57.90 (57.04, 58.76)	60.67 (59.88, 61.52)	59.08 (57.97, 60.06)	49.5	2.77	1.18
		4	65.29 (64.22, 66.37)	67.96 (66.87, 68.95)	67.04 (65.72, 68.13)	46.7	2.66	1.74
		5	65.27 (64.39, 66.15)	67.97 (67.00, 68.84)	67.00 (65.73, 68.10)	42.9	2.70	1.74
		6	66.97 (66.06, 67.84)	69.86 (68.99, 70.70)	68.26 (67.25, 69.52)	40.3	2.88	1.29
	70 (49)	1	70.93 (70.12, 71.74)	72.58 (71.39, 73.69)	72.76 (71.21, 74.21)	72.9	1.65	1.82
		2	76.73 (76.05, 77.58)	78.09 (77.20, 78.98)	78.35 (77.30, 79.55)	63.8	1.36	1.62
		3	57.80 (57.25, 58.39)	60.64 (60.03, 61.19)	58.88 (58.13, 59.65)	57.5	2.84	1.08
		4	65.24 (64.44, 66.02)	67.84 (67.13, 68.59)	66.73 (65.82, 67.64)	58.3	2.60	1.49
		5	65.20 (64.60, 65.84)	67.88 (67.21, 68.49)	66.81 (65.97, 67.63)	47.8	2.68	1.61
		6	67.06 (66.43, 67.66)	69.90 (69.28, 70.45)	68.23 (67.38, 69.16)	47	2.84	1.17
	199 (140)	1	70.74 (70.32, 71.24)	72.33 (71.61, 72.94)	72.43 (71.61, 73.39)	85.7	1.59	1.69
		2	76.87 (76.37, 77.33)	78.00 (77.36, 78.62)	78.51 (77.79, 79.27)	81.1	1.13	1.63
		3	57.84 (57.52, 58.17)	60.66 (60.32, 60.96)	58.78 (58.33, 59.21)	64.2	2.83	0.95
		4	65.23 (64.67, 65.71)	67.86 (67.41, 68.36)	66.78 (66.28, 67.29)	68.5	2.63	1.55
		5	65.21 (64.79, 65.59)	67.84 (67.39, 68.23)	66.69 (66.24, 67.24)	43.3	2.63	1.48
		6	67.13 (66.76, 67.47)	69.90 (69.58, 70.29)	68.18 (67.72, 68.69)	47.5	2.77	1.06
40-300	35 (24)	1	71.54 (70.70, 72.44)	72.31 (71.06, 73.61)	71.84 (70.63, 73.08)	50.4	0.77	0.30
		2	77.12 (76.29, 77.97)	77.79 (76.79, 78.81)	77.56 (76.20, 78.52)	45.1	0.67	0.44
		3	57.99 (57.36, 58.64)	59.95 (59.32, 60.58)	58.94 (58.09, 59.78)	34.9	1.96	0.94
		4	65.63 (64.80, 66.39)	67.23 (66.39, 68.00)	66.71 (65.73, 67.56)	37.2	1.60	1.08
		5	65.57 (64.83, 66.31)	67.19 (66.47, 67.91)	66.67 (65.70, 67.75)	32.4	1.62	1.11
		6	67.42 (66.70, 68.08)	69.15 (68.48, 69.85)	68.14 (67.18, 69.13)	32.5	1.73	0.71
	70 (48)	1	71.35 (70.72, 71.94)	72.00 (71.05, 72.88)	71.71 (70.73, 72.84)	69.2	0.65	0.36
		2	77.15 (76.59, 77.75)	77.84 (77.02, 78.61)	77.65 (76.79, 78.53)	66.1	0.68	0.49
		3	58.03 (57.56, 58.45)	60.02 (59.58, 60.46)	58.77 (58.26, 59.23)	41.1	1.99	0.74
		4	65.67 (65.05, 66.19)	67.26 (66.70, 67.82)	66.77 (66.11, 67.54)	45.2	1.59	1.10
		5	65.65 (65.14, 66.14)	67.24 (66.76, 67.76)	66.56 (65.96, 67.28)	37.7	1.59	0.91
		6	67.42 (66.87, 67.94)	69.14 (68.69, 69.59)	68.09 (67.26, 68.71)	40.4	1.72	0.67
	200 (139)	1	71.23 (70.89, 71.64)	71.91 (71.35, 72.48)	71.78 (71.13, 72.42)	86	0.68	0.55
		2	77.21 (76.85, 77.56)	77.91 (77.41, 78.39)	77.75 (77.24, 78.36)	80.4	0.70	0.55
		3	57.98 (57.74, 58.26)	59.99 (59.72, 60.23)	58.64 (58.28, 59.01)	46.3	2.01	0.66
		4	65.57 (65.23, 65.96)	67.22 (66.88, 67.57)	66.71 (66.30, 67.09)	44.2	1.65	1.15
		5	65.58 (65.27, 65.86)	67.20 (66.90, 67.49)	66.44 (65.99, 66.85)	34.6	1.62	0.86
		6	67.41 (67.12, 67.72)	69.13 (68.86, 69.44)	68.00 (67.62, 68.39)	39.1	1.72	0.59

B denotes the scenarios of biomarker coresponding to Table 2; CR shows convergence rate of the proposed method; estimates are summarized by median (first quantile, third quantiles); RB denotes reporting bias; Bias denotes bias of the proposed method.
